# Gene copy number variation and its significance in cyanobacterial phylogeny

**DOI:** 10.1186/1471-2180-12-177

**Published:** 2012-08-15

**Authors:** Bettina E Schirrmeister, Daniel A Dalquen, Maria Anisimova, Homayoun C Bagheri

**Affiliations:** 1 Institute of Evolutionary Biology and Environmental Studies, University of Zurich, Winterthurerstr. 190, 8057 Zurich, Switzerland; 2 School of Earth Sciences, University of Bristol, Wills Memorial Building, Queens Road, Bristol BS8 1RJ, UK; 3 Computational Biochemistry Research Group, Swiss Federal Institute of Technology, Universitätstrasse 6, 8092 Zurich, Switzerland; 4, Swiss Institute of Bioinformatics, Universitätstrasse 6, 8092 Zurich, Switzerland

**Keywords:** Prokaryotic phylogenetics, Concerted evolution, Gene copy number variation, Ribosomal rRNA, Cyanobacterial morphology, *Gloeobacter violaceus*

## Abstract

**Background:**

In eukaryotes, variation in gene copy numbers is often associated with deleterious effects, but may also have positive effects. For prokaryotes, studies on gene copy number variation are rare. Previous studies have suggested that high numbers of rRNA gene copies can be advantageous in environments with changing resource availability, but further association of gene copies and phenotypic traits are not documented. We used one of the morphologically most diverse prokaryotic phyla to test whether numbers of gene copies are associated with levels of cell differentiation.

**Results:**

We implemented a search algorithm that identified 44 genes with highly conserved copies across 22 fully sequenced cyanobacterial taxa. For two very basal cyanobacterial species, *Gloeobacter violaceus* and a thermophilic *Synechococcus* species, distinct phylogenetic positions previously found were supported by identical protein coding gene copy numbers. Furthermore, we found that increased ribosomal gene copy numbers showed a strong correlation to cyanobacteria capable of terminal cell differentiation. Additionally, we detected extremely low variation of 16S rRNA sequence copies within the cyanobacteria. We compared our results for 16S rRNA to three other eubacterial phyla (Chroroflexi, Spirochaetes and Bacteroidetes). Based on Bayesian phylogenetic inference and the comparisons of genetic distances, we could confirm that cyanobacterial 16S rRNA paralogs and orthologs show significantly stronger conservation than found in other eubacterial phyla.

**Conclusions:**

A higher number of ribosomal operons could potentially provide an advantage to terminally differentiated cyanobacteria. Furthermore, we suggest that 16S rRNA gene copies in cyanobacteria are homogenized by both concerted evolution and purifying selection. In addition, the small ribosomal subunit in cyanobacteria appears to evolve at extraordinary slow evolutionary rates, an observation that has been made previously for morphological characteristics of cyanobacteria.

## Background

Many genes originated via gene duplication in both prokaryotes and eukaryotes. Evolution after gene duplication can follow several scenarios [1]. Subfunctionalization leads to gene copies evolving specialized functions, all of which are necessary for performing the original gene function. In the neofunctionalization scenario, one gene copy is preserved by purifying selection, while the other copy may evolve a novel function through rapid adaptation. Finally, in a process known as pseudogenization, one gene copy will lose its function due to accumulation of mutations. Another possible evolutionary fate for gene duplicates is gene conservation. Conserved gene copies can be easily detected based on their high levels of sequence similarity, which typically occurs for genes whose products are needed in high concentrations. All gene copies are strongly expressed in such cases. Gene duplicates can maintain their identical function in two ways: by purifying selection which prevents the duplicates from diverging, or alternatively through concerted evolution where frequent gene conversion maintains sequence identity within the genome [1].

Gene copy number variants have been frequently found and studied in humans [2], but are also known to exist in other eukaryotic organisms, such as mouse [3], maize [4], and yeast [5]. Studies on human copy number variants revealed that multiple gene copies are often associated with diseases [6,7], but can also have positive effects as has been shown for salivary amylase genes [8]. Less is known about consequences of protein coding gene copy number variations in prokaryotes. Though there have been studies on variation of ribosomal RNA gene copy numbers and possible consequences [9,10]. Bacteria exhibiting multiple rRNA gene copies seem to respond faster to resource availability [11]. Accelerated growth rate has been conjectured to be a result of high ribosomal copy numbers [12]. In *E. coli* it is known that more than one rRNA operon has to be functional to express sufficient ribosomes and achieve maximum growth. Bacteria generally possess fewer than 10 rRNA gene copies [13], though some *Proteobacteria* and *Firmicutes* may have as many as 15 copies of rRNA operons [10]. Furthermore, ribosomal RNA copy numbers have been suggested to be phylogentically informative [14]. Phylogenetic positions of organisms and the amount of rRNA operon copy numbers they possess are generally associated.

Although potentially important effects of ribosomal copy numbers have been suggested in various studies, protein coding gene copies are less considered. This could be due to the assumption that selection for faster cell replication leads to genome reduction in prokaryotes [15], which would reduce the likelihood of survival of multiple gene copies. Indeed, a tendency towards genome reduction has been observed in endosymbiotic bacteria, and in free living prokaryotes including unicellular marine cyanobacteria [16]. However, conclusions that contradict this have been made by Kou and colleagues [17] who suggest that a lack of large prokaryotic genomes could be the result of selection acting on an upper limit of genome size. Thus, if there is no selective genome reduction in prokaryotes, multiple gene copies might be more widely distributed and of greater importance for prokaryotes than is believed so far.

Among prokaryotes cyanobacteria depict one of the morphologically most diverse phyla. Several of their morphotypes seem to exist for over two billion years as indicated by a well preserved fossil record [18,19]. Cyanobacteria inhabit diverse environments. They had (and still have) an exceptional influence on the planet due to their ability to conduct oxygenic photosynthesis and fix nitrogen. According to their morphology, cyanobacteria have been classified into five different sections [20], though molecular data indicate that probably none of the five groups is monophyletic [21-26]. Section I and II consist of unicellular cyanobacteria. Section II species can be distinguished from all other cyanobacteria based on their reproduction via multiple fission. Cyanobacteria belonging to section III to V exhibit filamentous growth. Across the five existing morphotype sections cyanobacteria exhibit several patterns of differentiation. The majority of extant cyanobacterial species control gene expression using a circadian clock. Additionally, several multicellular cyanobacteria developed mechanisms to differentiate not only temporarily, but also spatially. *Trichodesmium* is the only section III genus known, able to produce specialized cells (‘diazocytes’) in the middle of a filament [27-29]. The principal form of terminal cell differentiation is observed in section IV and V cyanobacteria. Given the morphological variety found in this phylum, we ask whether gene dosage (multiple gene copies per cell) is associated with adaptive morphological strategies such as cell differentiation in cyanobacteria. Variation in 16S rRNA gene copy sequences and numbers has been reported previously for cyanobacterial genera [30,31], but no phenotypic correlations were found. Little is known about protein coding gene copy numbers in cyanobacteria.

In this study we searched for both ribosomal RNA and protein coding gene copy number variation in diverse species of cyanobacteria for which full genome sequences were available. Ribosomal RNA gene copies were examined since it is known that they might occur in multiple copies and exhibit gene dosage effects [11-13]. Segments of genes within the rRNA operon are strongly conserved because of their functional relevance [32]. These unique features have made 16S rRNA gene sequences a favored taxonomic marker for prokaryotes [33]. Although rRNA sequence variation within a genome is low for most species [9], considerable intragenomic differences have been reported in some non-cyanobacterial species [10,34]. This has led to the questioning of the reliability of 16S rRNA genes as a taxonomic marker. We examined sequence identity of rRNA genes within species of cyanobacteria by conducting phylogenetic analyses and calculating phylogenetic distances. Results for cyanobacteria were compared to data from the prokaryotic phyla Chroroflexi, Spirochaetes, and Bacteroidetes. Paralogs of 16S rRNA genes are almost identical in cyanobacterial species and suggest a deviation from divergent evolution of gene copies. Investigating variation in copies of the internal transcribed spacer region (ITS), located between the 16S and 23S rRNA genes, suggests that both concerted evolution and purifying selection are viable hypotheses for the evolution of 16S rRNA in cyanobacteria. Furthermore, we observed an exceptionally strong sequence conservation in 16S rRNA orthologs within the cyanobacterial phylum. A level of conservation that could not be observed in any of the eubacterial phyla studied here.

## Results and discussion

### Identification of conserved gene copies and their phylogenetic relevance

Aside from ribosomal RNA genes, we identified 41 protein coding genes which possess multiple conserved gene copies in at least one cyanobacterial species (Additional file 1). From this total of 44 genes, only six showed significant correlations to morphological characteristics. Ribosomal RNA genes were the main class of genes exhibiting conserved gene copies that were significantly correlated to the cyanobacterial sections IV and V. Species capable of terminal cell differentiation exhibited four or five copies of ribosomal genes. Furthermore, *Gloebacter violaceus* and a thermophilic *Synechococcus* species share a distinct pattern of gene copy numbers which adds independent support to previous studies that have grouped these species separately from the rest of cyanobacteria, closer to an eubacterial outgroup [22,35-39].

**Figure 1:**
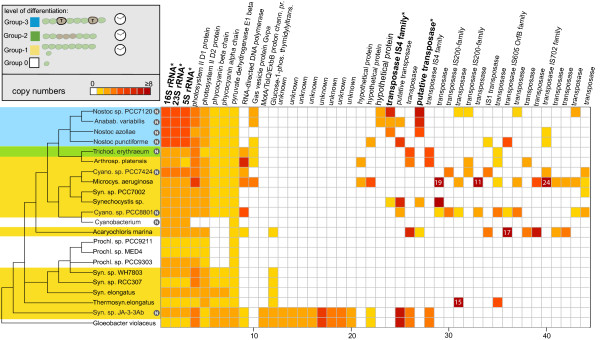
**Conserved paralogs in cyanobacteria.** Distribution of gene copy numbers within and across cyanobacterial genomes. On the left side cyanobacterial cladogram is shown, emphasizing the different morphological groups. Species of group G1 exhibiting circadian rhythm are displayed in a yellow box. *Trichodesmium* exhibiting reversible differentiation is shown in a green box (group G2) and cyanobacteria of group G3 which are able to terminally differentiate, are displayed in a blue box. The letter ‘N’ marks species capable of nitrogen fixation. Conserved copy numbers of genes are shown in a color plot ranging from yellow indicating a single gene to dark red denoting 8 copies or more. In cases where gene copy numbers exceed 8, values are given in white letters. Corresponding species names are written on the left and gene names are written on top. Copy numbers of genes displayed in bold and marked by a “*” are positively correlated to terminal differentiation.

We investigated conserved gene copies that exhibited ≥90*%*(not shown), ≥95*%*(not shown) and ≥98*%* amino acid sequence identity within a genome. Results varied mainly in numbers of transposase gene copies detected. Therefore, results of gene copies with an identity of ≥98*%*within a genome and ≥50*%*between species are presented here. For these genes, we mapped copy numbers in relation to the phylogenetic position within cyanobacteria (Figure 1). The highest number of gene copies (24) was found for a transposase encoding gene in *Microcystis aeruginosa*. Transposases are enzymes that catalyze the movement of transposable elements. Previous studies have estimated that genes encoding for transposases are the most widespread genes, and often occur as multiple copies [40]. Almost half of the conserved gene copies identified in this study were transposase encoding genes. The frequency of transposase genes varied between different species. *Microcystis aeruginosa* possessed various transposase genes, whereas strains belonging to the genera Synechococcus and Prochlorococcus, and *Cyanobacterium sp. UCYN-A* seem to exhibited fewer transposase gene copies.

*Synechococcus sp. JA-3-3Ab*, a unicellular cyanobacterium isolated from a hot spring in Yellow Stone National Park [41,42], exhibited a pattern of gene copy numbers that generally deviated from the pattern observed in other *Synechococci*. It shared identical copy numbers of protein coding genes with *Gloeobacter violaceus*. These included a series of not yet annotated genes missing in all other cyanobacteria. This pattern of almost identical conserved gene copy numbers supports other phylogenetic and phylogenomic studies that place these two species close to each other at the base of the cyanobacterial phylogenetic tree [36-38]. In a previous study using 16S rRNA sequences, Schirrmeister *et al.*[39] observed a close phylogenetic relationship of *Gloeobacter violaceus* and another Synechococcus strain [43] isolated from the same source as *Synechococcus sp. JA-3-3Ab*. Similar results have been found elsewhere [22]. The phylogenetic distance of *Gloeobacter violaceus* to other extant cyanobacteria has been pointed out before [35]. Major differences involve the light harvesting machinery. *Gloebacter violaceus* lacks thylacoid membranes [44], and various genes from photosystems I and II.

**Figure 2:**
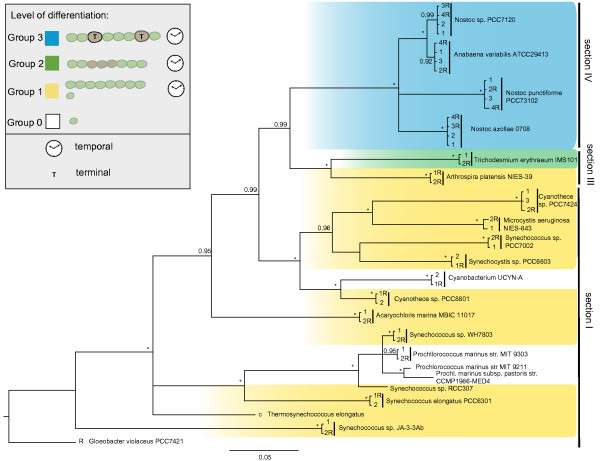
**Cyanobacterial tree including all 16S rRNA gene copies.** Cyanobacterial tree including all 16S rRNA copies, reconstructed using Bayesian analysis. Posterior probabilities >0.90 are displayed on the nodes. Colors indicate species-groups according to differentiation level. Species in yellow boxes control gene expression only via a circadian rhythm. Genus *Trichodesmium* shown in a green box is able to produce temporarily differentiated cells, called ‘diacocytes’. Multicellular species able to form terminally differentiated cells are shown in blue boxes. The letter “R” denotes gene copies that are positioned on the reverse DNA strand. Multicellular, terminally differentiated cyanobacteria are the only species exhibiting four copy numbers. Regardless of morphology, 16S rRNA sequences are highly conserved within each genome.

Furthermore, we identified several genomes with more than one ribosomal gene copies. Cyanobacterial taxa used in this study exhibited one to four conserved rRNA gene copies (Figure 1, Table 1). Position of ribosomal gene copy numbers across the Bayesian tree were phylogenetically non-informative (Figures 1 and 2). However, four rRNA copies could only be observed in terminally differentiated species. Additional data on 16S rRNA copy numbers shown in the rrn-database, confirmed these findings and furthermore reported five copies for several cyanobacterial species belonging to sections IV and V. Aside from 16S rRNA data, no further information was obtained, because these taxa have not been fully sequenced, yet [45].

**Table 1:** Data of cyanobacterial 16S rRNA gene sequences

**Species**	**Group**	**Genome size**	**# of copies**	**d^1 ^**	**F**	**F**	**R**	**R**	**Accession nr.**
*Acharyochloris marina* MBIC11017	G1	8.36	2	0	5,636,175		1,409,149		CP000828.1
*Anabaena variabilis* ATCC 29413	G3	7.10	4	0	1,002,918	3,894,075	2,808,379	5,435,874	CP000117.1
*Arthrospira platensi*s NIES 39	G1	6.80	2	0			2,584,861	3,509,612	AP011615
*Cyanothece sp.* PCC 7424	G1	6.52	3	0.001	1,328,842	3,465,297	2,494,023		CP001291.1
*Cyanothece sp.* PCC 8801	G1	4.81	2	0	3,806,018		2,484,826		CP001287.1
*Gloeobacter violaceus* PCC 7421	G0	4.70	1				1,571,231		BA000045.2
*Microcystis aeruginosa* NIES-843	G1	5.80	2	0.003	1,885,807		3,597,272		AP009552.1
*Nostoc azollae* 0708	G3	5.53	4	0	830,919	2,217,271	979,079	2,979,417	CP002059.1
*Nostoc punctiforme* PCC 73102	G3	9.01	4	0.002	2,021,489	6,085,170	5,515,629	6,502,973	CP001037.1
*Nostoc sp.* PCC 7120	G3	7.20	4	0	2,375,734	2,500,525	4,918,283	5,945,700	BA000019.2
*Prochlorococcus marinus* MIT 9211	G0	1.70	1		342,283				CP000878.1
*Prochlorococcus marinus* MIT 9303	G0	2.70	2	0	243,682		1,938,786		CP000554.1
*P. marinus subsp. pastoris* str. CCMP1986 (MED)	G0	1.70	1		313,061				BX548174.1
*Synechococcus elongatus* PCC 6301	G1	2.70	2	0	1,656,455		1,050,801		AP008231.1
*Synechococcus sp.* JA-3-3Ab	G1	2.90	2	0	2,310,397		1,110,127		CP000239.1
*Synechococcus sp.* PCC 7002	G1	3.40	2	0	1,461,361		2,909,371		CP000951.1
*Synechococcus sp.* RCC307	G1	2.20	1		348,765				CT978603.1
*Synechococcus sp.* WH 7803	G1	2.40	2	0	534,563		2,019,450		CT971583.1
*Synechocystis sp.* PCC 6803	G1	3.97	2	0	3,325,053		245,2187		BA000022.2
*Thermosynechococcus elongatus* BP-1	G1	2.59	1				2,335,243		BA000039.2
*Trichodesmium erythraeum* IMS101	G2	7.80	2	0	3,137,164		4,601,878		CP000393.1
*Cyanobacterium* UCYN-A	G0	1.40	2	0	638,681		3,507		CP001842.1

d^1^: Largest distance between gene copies within the genome. F: Coordinates for the 16S rRNA genes on the forward strand of the chromosome. R: Coordinates for the 16S rRNA genes on the reverse strand of the chromosome.

### Correlation of copy numbers to terminal differentiation

**Figure 3:**
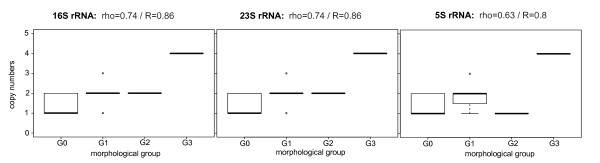
**Dispersion of gene copy numbers in different groups of differentiation.** A boxplot representation of the gene copy number dispersion across the previously defined morphological groups. Shown are dispersions for genes from the rRNA operon. Spearman’s rank correlation coefficient (*ρ*) and Pearson’s correlation coefficient (R) are displayed above the corresponding graph. Positive correlation coefficients of rRNA gene copies to terminally differentiated cyanobacteria are supported.

To confirm possible associations of ribosomal RNA copy numbers to species capable of terminal cell differentiation, we visualized the distribution of ribosomal gene copy numbers and tested for possible correlations to morphotypes (Figure 3). We additionally calculated potential correlations of all protein coding gene copy numbers identified in this study with morphotypes. Therefore, we divided cyanobacteria into four morphological groups according to their mode of differentiation. Group 0 (G0) exhibits no mode of differentiation and contains solely unicellular species. Group 1 (G1) consists of species from section I to III which control gene expression via a circadian rhythm, but lack any other form of differentiation. Group 2 (G2) is formed exclusively by the genus *Trichodesmium* which is able to form temporarily differentiated cells for nitrogen fixation. The last group (G3) contains species from section IV and V which are able to produce terminally differentiated cells.

Using Spearman’s rank correlation coefficient (*ρ*) and Pearson’s correlation coefficient (R), we estimated a potential correlation of copy numbers to the defined morphological groups. Both tests indicated significant correlations to morphological groups for all ribosomal genes and two transposase coding genes. Furthermore, Spearman’s *ρ*attested a significant correlation to morphology for photosystem II reaction center D2 protein (*ρ*=0.62), and a weaker correlation to Gas vesicle protein GVPa (*ρ*=0.58) coding genes. A significant Pearson’s correlation was found for a gene coding for a hypothetical protein (*R*=0.58). In Figure 3 distributions of ribosomal RNA gene copy numbers across morphological groups are presented as boxplot graphics with correlation coefficients, and p-values shown. All taxa capable of terminal differentiation exhibited four copies of ribosomal RNA genes. Correlation coefficients for 16S and 23S rRNA genes were *ρ*=0.74/R=0.86, in both cases, and *ρ*=0.63/R=0.8 for the 5S rRNA genes. Including additional data from the rrn-database [45] (Additional file 2), resulted in an even stronger correlation of 16S rRNA gene copy numbers to cyanobacterial species capable of terminal differentiation (*ρ*=0.87/*R*=0.9; Additional file 3). Cyanobacteria belonging to section IV and V form terminally differentiated cells (called heterocysts) in the absence of fixed nitrogen. In these cells oxygen sensitive nitrogen fixation can take place while neighbouring cells conduct oxygenic photosynthesis. These heterocystous cells undergo various structural and physiological alterations to protect nitrogenase from oxygen in a ‘microanaerobic’ environment. As a result they lose their ability to conduct photosynthesis and to divide. Multiple rRNA gene copies could have positive effects during heterocyst formation, the same way as they help *E.coli* to achieve maximum growth [12], and increases responses to changing environmental conditions [11]. An increased amount of functional ribosomal operons likely depicts an advantage in the process of cell differentiation, during which expression of various genes is upregulated [46].

### Strong conservation of 16S rRNA copies

Previous studies have sometimes questioned the potential of 16S rRNA gene sequences as a taxonomic marker due to variation that has been observed between gene paralogs in some non-cyanobacterial organism [10,34]. We explored sequence variation of 16S rRNA genes in cyanobacteria by reconstructing phylogenetic trees with Bayesian inference. We evaluated the divergence of 16S rRNA gene copies within and between cyanobacterial taxa. The inferred Bayesian consensus tree is displayed in Figure 2. Investigated cyanobacteria, exhibit one to four 16S rRNA copies per genome. Unicellular species partition in two major groups: species belonging to the marine pico-phytoplankton genera *Synechococcus* and *Prochlorococcus*, and members of the genera *Synechocystis*, *Cyanothece* and *Microcystis* which show a closer relation to multicellular cyanobacteria. All multicellular species studied here are closely related, and species capable of terminal differentiation form a monophyletic group. Comparisons of our study to previous findings show high similarities. Our results agree with a comparative phylogenomics approach used by Swingley *et al.*[36], a consensus tree of concatenated sequences presented by Blank and Sànchez-Baracaldo [47], and, are highly similar to 16S rRNA analyses conducted by Schirrmeister *et al.*[39]. Using a larger taxon set [39], we previously inferred polyphyletic groupings of undifferentiated multicellular species belonging to section III. This however is not deducible from the taxonomically more limited full genome data set used in the present study.

**Figure 4:**
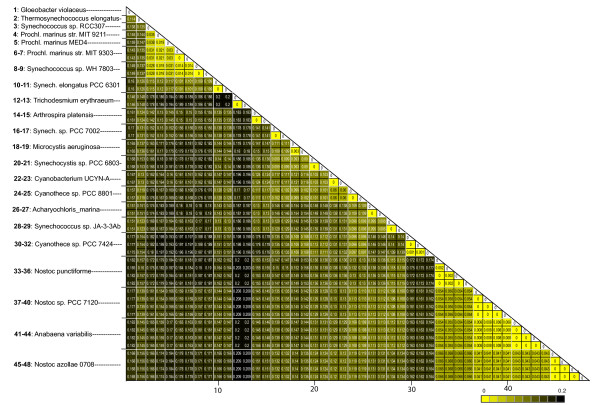
**Distance matrix of cyanobacterial 16S rRNA sequences.** Distance matrix between 16S rRNA genes estimated based on K80 substitution model. 16S rRNA gene copy numbers range from one to four per cyanobacterial genomes studied. White lines separate sequence copies of different species. 16S rRNA sequences are highly conserved within species.

In cyanobacteria 16S rRNA sequences were highly conserved within a genome. Three species showed minor nucleotide differences. The two 16S rRNA copies of *Microcystis aeruginosa* differed by four ‘single nucleotide polymorphisms’ (SNPs), in *Cyanothece sp.* PCC 7424 one SNP was detected, and in *Nostocpunctiforme* one 16S copy possessed two SNPs. The differences are visualized in a molecular distance matrix in Figure 4. 16S rRNA copies within species were identical for the majority of taxa (shown in yellow) and can be clearly distinguished from gene copies belonging to different species. Furthermore, using the whole dataset we calculated mean distances within strains (*d*_*W*_) and between strains (*d*_*B*_). Results are presented in Table 2. Significance of differences in sequence distances found within and between cyanobacterial strains were estimated using bootstrap re-sampling of the original data set. Distributions of the resulting mean distances are displayed in Additional files 4 and 5. For each distribution, an overall mean distance was calculated ( $d_{W}^{*}$$d_{B}^{*}$). Mean distance of 16S rRNA sequences within species (*d*_*W*_=0.0001) is significantly smaller than between species (*d*_*B*_=0.14; Table 2). 95% confidence intervals of distributions obtained by re-samplings do not overlap. Although previous studies have claimed that variation within 16S rRNA sequences might affect reliability of this gene as a taxonomic marker [10,34], this was not found for genera used in this study. Rather, the extreme sequence conservation of 16S rRNA gene copies from the same species supports 16S rRNA as a reliable genetic marker for the taxa analyzed here.

**Table 2:** Comparison of mean distances within cyanobacteria and to other eubacterial phyla

**Within a genome**				
	***d*_*W*_**	$d_{W}^{*}$	**95% confidence intervals**	
			***lower***	***upper***
Cyanobacteria	0.0001	0.0003	0.0001	0.0005
Chloroflexi	0.0036	0.0020	0.0012	0.0028
Spirochaetes	0.0012	0.0009	0.0005	0.0014
Bacteroidetes	0.0029	0.0023	0.014	0.0032
**Between species**				
	***d*_*B*_**	$d_{B}^{*}$	**95% confidence intervals**	
			***lower***	***upper***
Cyanobacteria	0.1427	0.1426	0.1235	0.1587
Chloroflexi	0.3409	0.434	0.2489	0.4087
Spirochaetes	0.3537	0.3541	0.2907	0.4017
Bacteroidetes	0.3779	0.378	0.3390	0.4099

Comparison of mean distances in the different eubacterial phyla and the 95% confidence intervals of 10,000 mean values calculated from bootstrap samples. Confidence intervals do not overlap between cyanobacteria and the other eubacterial phyla. Distances of 16S rRNA sequences are significantly smaller in cyanobacteria compared to the other prokaryotes.*d*_*W*_ and *d*_*B*_: mean calculated from the original dataset including all distances. $d_{B}^{*}$ and $d_{W}^{*}$: mean of 10,000 means calculated using bootstrap sampling.

**Figure 5:**
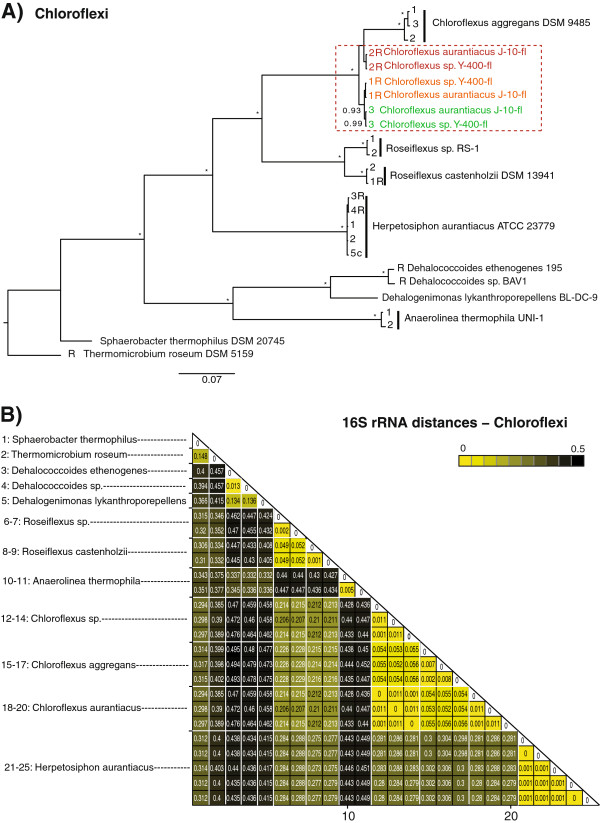
**Phylogenetic tree and distance matrix of Chloroflexi including all 16S rRNA copies.** (**A**) Phylogenetic tree of the eubacterial phylum Chloroflexi including all 16S rRNA copies, reconstructed using Bayesian analysis. On the nodes posterior probabilities >0.90 are displayed. Colored taxa mark species where 16S rRNA copy numbers evolved rather via divergent evolution, than being homogenized within a strain via concerted evolution. The letter “R” denote gene copies that are positioned on the reverse DNA strand. (**B**) Distance matrix of Chloroflexi. Genetic distances have been estimated according to the K80 substitution model. White lines separate sequence copies of different species. 16S rRNA sequences are conserved within species, but exhibit more variation than found for cyanobacteria.

In order to verify the significance of our results for cyanobacteria, we compared phylogenetic and distance results from the cyanobacteria to three eubacterial phyla (Chroroflexi, Spirochaetes and Bacteroidetes). Figure 5 presents the Bayesian consensus phylogenetic tree and the distance matrix reconstructed for the phylum Chloroflexi. Trees and distance matrices for the phyla Spirochaetes, and Bacteroidetes are shown in Additional files 6, 7 and 8. Within the phylum Chloroflexi, species contain one to five 16S rRNA genes per genome. The phylogenetic tree is well supported by posterior probabilities. Previous phylogenetic studies have divided the phylum Chlorophlexi into several subdivisions [48,49], the majority of which is supported by our inferred tree. Distances of the 16S rRNA sequences within genomes and between species of Chloroflexi were significantly higher than found for cyanobacteria (Table 2). Mean distances of species belonging to the phylum Chloroflexi were *d*_*W*_=0.004 within species, and showed a 10-fold difference compared to distances between species (*d*_*B*_=0.34). *Chloroflexus auranticus* and *Chloroflexus sp.* were the only species among the taxa analyzed in this study where 16S rRNA orthologs were more similar than their paralogs. Further comparison of mean distances for 16S rRNA sequences including phyla Spirochaetes and Bacteroidetes confirmed the significantly lower sequence variation in cyanobacteria. A comparison of the distributions of mean distances calculated from the bootstrap re-sampling show no overlap of the 95% confidence intervals of cyanobacteria and any of the other phyla (Additional files 4 and 5). Furthermore, within all studied phyla, mean distances for 16S rRNA gene copies within a genome (*d*_*W*_) were smaller by at least one order of magnitude compared to mean distances for 16S rRNA sequences between species (*d*_*B*_). Our results support 16S rRNA as an adequate taxonomic marker for the species analyzed in this study and agree with previous findings of limited heterogeneity in 16S rRNA [9].

### Evolution of 16S rRNA gene copies in cyanobacteria

**Figure 6:**
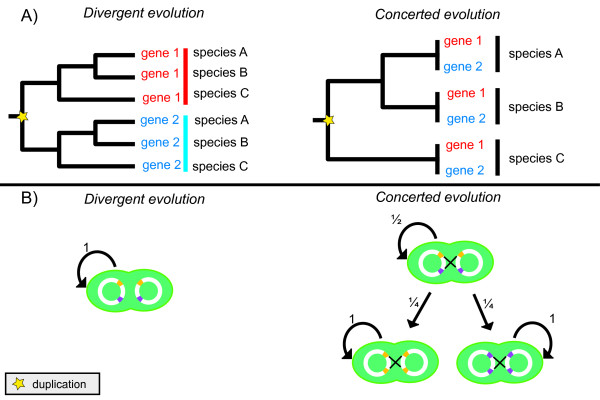
**Divergent and concerted evolution.** (**A**) The phylogenetic pattern of divergent and concerted evolution evolution. Paralogs and orthologs diverge at similar degrees in the first scenario, while they get frequently homogenized during concerted evolution. A cyanobacterial cell during cell division without homologous recombination. All daughter cells will exhibit the same chromosome as the mother cell. (**B**) Replication pattern during cell division under divergent and concerted evolution. If during cell devision homologous recombination takes place in half of the recombinants the daughter cells will exhibit the same chromosome as the mother. For the other half of recombinants, each gene copy has a $\frac{1}{4}$ chance of replacing the other. Once gene copies are identical homologous recombination cannot reverse the process. Hence if this process is repeated recursively at a population level, one gene copy will eventually get fixed.

Two mechanisms may conserve sequences of gene copies: purifying selection and concerted evolution. These two can be distinguished by examining variation patterns in non-coding regions [1,50]. In the case of purifying selection, non-coding regions are thought to evolve neutrally, accumulating mutations over time due to genetic drift. If concerted evolution shapes gene copies, the entire gene sequence including non-coding regions and synonymous sites are homogenized. During this process, genes evolve in ‘concert’, which is commonly observed in plants and fungi [51,52] (Figure 6). Subsequently, paralogs show stronger similarities than orthologs, as a result of intragenomic homologous recombination [53].

The strong conservation of 16S rRNA sequence copies in cyanobacteria and Eubacteria examined here suggests that 16S rRNA in these species is shaped by strong purifying selection and/or concerted evolution. Generally, it is assumed that ribosomal genes in Archaea and Eubacteria are shaped by concerted evolution [13]. 16S rRNA genes can be subdivided in strongly conserved and more variable regions. One would expect that if purifying selection acts as the major force for conservation of gene copies within a genome, some neutral variation should be detected in these variable regions. The extraordinary conservation of 16S rRNA in cyanobacteria seems to indicate that concerted evolution is a more likely explanation. To verify this suggestion we examined variation in the internal transcribed spacer region, located between the 16S and 23S rRNA gene. Though previous studies have suggested conservation of some regions in the ITS sequence, several regions should not be affected by selection and evolve neutrally. If the entire ITS sequence showed the same degree of conservation as does the 16S gene sequence, then purifying selection —which would only act on the functional parts— could be rejected as a driving force. However, the strong conservation found in cyanobacterial 16S rRNA gene sequences could not be confirmed for the ITS-regions of four cyanobacterial taxa (Additional file 9). For cyanobacteria and the eubacterial phyla studied here, both concerted evolution and strong purifying selection, appear to be the main contributing factors.

Although, cyanobacteria are assumed to be an ancient phylum which presumably raised oxygen levels in the atmosphere more than 2.3 billion years ago [54], variation in 16S rRNA copies is extremely low. Indeed, phylogenetic tree reconstructions for 16S rRNA result in relatively short estimated branch lengths within this phylum, compared to other eubacterial phyla (Figure 2). Short evolutionary distances for 16S rRNA sequences are consistent with a pattern that has been found for morphological characters in cyanobacteria before. In 1994, J.W. Schopf compared the tempo and mode of evolution in cyanobacteria from the Precambrian, to evolutionary patterns observed in fossils during the Phanerozoic. The latter have been described by G.G. Simpson in his book “The tempo and mode of evolution” [55]. Schopf found that evolutionary predictions which Simpson made for metazoan fossils from the Phanerozoic, can also be applied to cyanobacteria. Morphologically, cyanobacteria seem to evolve not only at a “bradytelic”, but “hypobradytelic” mode, meaning at exceedingly low evolutionary rates. Fossils from the Precambrian strongly resemble present morphotypes. The oldest undisputed cyanobacterial fossils date back circa 2.0 billion years [18,19]. Morphological appearance of these microfossils already suggests the presence of at least four of the morphological sections described by Castenholz [20]. It seems that cyanobacteria reached their maximum morphological complexity two billion years ago, and many of today’s species could be described as so-called ‘living fossils’. It remains to be seen whether the low evolutionary rates as seen in 16S rRNA sequences and morphological features, is also seen at the genomic and metabolic level. This question can be further resolved as further genomic sequences become available for the cyanobacteria.

## Conclusion

Among 22 fully sequenced cyanobacterial taxa that were carefully chosen according to phylogenetic position and morphological characteristics, we identified 41 protein coding genes that occur as multiple highly conserved copies in at least one cyanobacterial species. Copy numbers of ribosomal genes show a significant correlation to cyanobacterial species that are capable of terminal differentiation. The formation of heterocysts, morphologically modified cells for nitrogen fixation, requires a strong increase in gene expression, for which an accumulation of ribosomes could be of potential advantage. Further testing would be required though, to make causal conclusions for increased rRNA operons in cyanobacteria belonging to section IV and V. Furthermore, phylogenetic analyses revealed a high conservation of 16S rRNA copies within eubacterial species. Though this is true for all phyla that have been analyzed, cyanobacteria exhibit an exceptionally strong conservation. Comparison to variation in ITS regions point to concerted evolution via homologous recombination and purifying selection as the forces behind 16S rRNA sequence evolution. Comparison of interspecific genetic distances within several prokaryotic phyla, showed significantly lower variation of cyanobacterial 16S rRNA gene sequences. This suggests that 16S rRNA gene sequences evolve by a ‘hypobradytelic’ mode of evolution, previously suggested for morphological characteristics in cyanobacteria [56].

## Methods

### Data choice and description

For this study we only used cyanobacterial taxa with fully sequenced and annotated genomes publicly available on GenBank (*http://www.ncbi.nlm.nih.gov/genomes/lproks.cgi*). Of those 42 genomes (as of August 2011), 36 belong to singlecelled strains, covering 10 different species in total. The remaining six genomes belong to multicellular strains, each representing another species. The taxon sampling was done to exclude a bias towards unicellular closely related cyanobacteria which are overrepresented in the genome-database [57]. Therefore, to cover the widest possible range of morphotypes, we selected one or more, fully sequenced taxa of each species for a total dataset of 22 cyanobacterial strains. More precisely, we included multiple strains of species *Cyanothece sp.*(2), *Synechococcus sp.*(4), and *Prochlorococcus marinus*(3), which, following the examination of previous phylogenies [39,47,58,59], are assumed to add phylogenetic diversity. No outgroup was included in the phylogenetic analyses. *Gloeobacter violceus* has been shown to be closest to eubacterial outgroups [39]. Therefore, phylogenetic trees are represented accordingly.

### Identification of conserved paralogs and correlation to morphotypes

In order to find genes with multiple copies, we applied the orthology prediction algorithm OMA [60] to the set of 22 complete cyanobacteria genomes. First we looked for clusters of highly conserved paralogous genes within each species. From the all-against-all pairwise sequence alignments computed by OMA, we selected pairwise hits within each species with an alignment score of at least 130 and minimum sequence identity of ≥98*%*, ≥95*%* and ≥90*%*. We then used these hits as edges in a homology graph, and identified clusters of highly conserved paralogs as connected components. Finally, we removed hits within a cluster if the pairwise distance differed significantly from the mean distance within the cluster. In the second step, we grouped detected homologous clusters across species using OMA alignments, but this time with a score cut-off of 180 and minimum sequence identity of ≥50*%*. We further required that ≥0.8·*n*_*i*_·*n*_*j*_of hits between any pair of clusters *i* and *j* be present in order to be considered, where *n*_*i*_*n*_*j*_ is the number of genes in clusters *i* and *j*, respectively. If a cluster in one genome grouped with several clusters in another genome, we chose the one with the lowest average pairwise distance. Again, homologous groups were extracted as connected components from the resulting graph. Finally, single orthologs from the OMA orthologous matrix (i.e, with no detected multiple copies within their originating genome) were matched and added to corresponding homologous groups.

We tested whether a correlation between cell differentiation and copy numbers could be observed for the identified genes. To do this, we devided cyanobacterial species into four different groups of cell differentiation (G0-G3; see results). Five strains belong to G0, 12 taxa belong to G1, *Tricodesmium* is the only genus in G2, and four species belong to G3. For 16S rRNA genes additional data could be obtained from rrndb-database [45] (Additional file 3). Adding these data resulted in a taxon set of 16S rRNA gene sequences as follows: five strains belonging to G0, 12 strains representing G1, *Trichodesmium* as the only species in G2 and 11 species in G3. Spearman’s rank and Pearson’s correlation coefficients were applied in order to estimate associations between conserved copy numbers and morphological groups (G0-G3), using R-software. Correlations with a p-value<0.01 were considered to be significant.

### Phylogenetic analyses

We conducted separate phylogenetic analyses of 16S rRNA gene sequences of cyanobacteria (Table 1) and four different eubacterial phyla (Additional file 10). For all taxa included in the phylogenetic trees, full genome sequences were available. All sequences were downloaded from GenBank [61]. For cyanobacteria two phylogenetic trees were reconstructed. One including a single 16S rRNA sequence per taxon and another including all 16S rRNA copies per taxon. Final taxon sets included 22 sequences in the first case and 48 sequences in the latter. The datasets were aligned using Clustal-X software with default settings [62] (1,325nt incl. gaps). Gaps were excluded from the analysis. Phylogenetic reconstructions were done using Bayesian analysis as implemented in MrBayes software [63]. Two Metropolis coupled Markov Chain Monte Carlo (*MC*^3^) searches were run for 10^7^ generations each using three heated and one cold chain. Figure 1 and Figure 2 show the consensus trees of 16,002 trees that were sampled every 1,000th generation from the *MC*^3^ searches, excluding the first 2,000 trees of each run (burn-in). At that point the log probabilities reached stationarity and average standard deviation of split frequencies were below 0.02. Performance of the MCMC and stationarity of the parameters were checked using Tracer v1.5 [64]. Effective Sample Sizes (ESS) were all above 200, supporting a well mixed MCMC run.

Phylogenetic analysis described for cyanobacteria was equally conducted for the phyla Auificae, Bacteroidetes, Chloroflexi and Spirochaetes. The non-cyanobacterial phylogenetic trees were reconstructed including all 16S rRNA gene copies of each taxon. *MC*^3^analyses were run for 10^6^ generations. The first 200,000 generations of each run were discarded as a burn-in. Parameters and trees were sampled every 1,000th generation resulting in a final set of 1,602 trees. The resulting Bayesian consensus trees for each phylum with posterior probabilities displayed at the nodes, have been visualized with FigTree v1.3.1 [65].

### Molecular distance analyses

For each set of aligned 16S rRNA gene sequences, distance matrices were calculated applying a K80 substitution model as implemented in the program baseml of PAML v4.3 [66]. The same was done for the internal transcribed spacer region (ITS) in cyanobacteria (Additional file 9). The resulting numeric matrices were imaged as color matrices using the R-package “plotrix” [67]. The color gradient of each matrix was scaled by the matrix’s minimum and maximum values. Mean distances were calculated within strains (between paralogs; *d*_*W*_) and between strains (between orthologs; *d*_*B*_), for each phylum. Significant differences in mean distances were confirmed with bootstrap re-samplings of independent values from the original dataset. To estimate significant differences of mean distances within species (*d*_*W*_), independent distance values were sampled 10,000 times for each species. Bootstrap re-sampling was done on each of these sample sets. Mean distances were hence calculated and their distribution plotted in a histogram (Additional file 4). The resulting overall mean, $d_{W}^{*}$ of the distributions, as well as 95% confidence intervals are presented in Table 2. To confirm potential differences of mean distances between species (*d*_*B*_) compared to other phyla, independent values were sampled 10,000 times. These datasets were re-sampled and mean distances calculated. The distributions are displayed in Additional file 5. The resultant overall mean, $d_{B}^{*}$ of each distribution, as well as 95% confidence intervals are shown in Table 2. Independence of distance estimations was assumed if from the corresponding matrix each column and row was only chosen once.

## Competing interests

The authors declare that they have no competing interests.

## Authors’ contributions

BES and HCB conceived the study; BES gathered data; BES and DAD conducted analyses; BES, DAD, MA and HCB designed research and wrote the paper. All authors read and approved the final manuscript.

## Supplementary Material

Additional file 1 Identified gene copies. The sheet contains Information on 41 gene copies and their presence in 22 cyanobacterial species. Amino acid sequences of the coded proteins exhibit 98% similarity within a genome and 50% across species. Click here for file

Additional file 2 16S rRNA gene copy data including data from the rrndb-database. Table with information on 16S rRNA copy numbers including data received from the rrnDB database [45] marked (*). Click here for file

Additional file 3 Distribution of 16S rRNA copy numbers using additional data from rrndb3. Boxplot representations of the 16S rRNA gene copy number distribution across the previously defined morphological groups. Additional data on 16S rRNA copy numbers were received from the rrndb-database [45]. Spearman’s rank correlation coefficient (*ρ*) and Pearson’s correlation coefficient (R) are displayed above the graph. A strong correlation of 16S rRNA gene copies to terminally differentiated cyanobacteria is supported. Click here for file

Additional file 4 Distribution of mean distances within species of bootstrap samples for the different eubacterial phyla. The distribution of mean distances of the bootstrap samples presented as a histogram. The 95% confidence intervals between cyanobacteria and Chloroflexi, Spirochaetes and Bacteroidetes do not overlap. Cyanobacterial 16S rRNA gene sequence variation within species is significantly lower. Click here for file

Additional file 5 Distribution of mean distances between species of bootstrap samples for the different eubacterial phyla. The distribution of mean distances of the bootstrap samples presented as a histogram. The 95% confidence intervals between cyanobacteria and the other eubacterial phyla do not overlap. Cyanobacterial 16S rRNA gene sequence variation between species are significantly lower. Click here for file

Additional file 6 Phylogenetic tree and distance matrix of Spirochaetes. (**A**) Phylogenetic tree of the eubacterial phylum Spirochaetes including all 16S rRNA gene copies, reconstructed using Bayesian analysis. On the nodes posterior probabilities >0.90 are displayed. The letter “R” denote gene copies that are positioned on the reverse DNA strand. (**B**) Distance matrix of Spirochaetes. Genetic distances have been estimated according to the K80 substitution model. White lines separate sequence copies of different species. Click here for file

Additional file 7 Phylogenetic tree of Bacteroidetes. Phylogenetic tree of the eubacterial phylum Bacteroidetes including all 16S rRNA gene copies, reconstructed using Bayesian analysis. On the nodes posterior probabilities >0.90 are displayed.The letter “R” denote gene copies that are positioned on the reverse DNA strand. Click here for file

Additional file 8 Distance matrix of Bacteroidetes. Genetic distances have been estimated according to the K80 substitution model. White lines separate sequence copies of different species. Click here for file

Additional file 9 Distance matrix of cyanobacterial ITS-region. Distance matrix of the internal transcribed spacer sequence region in cyanobacteria. Genetic distances have been estimated according to the K80 substitution model. White lines separate sequence copies of different species. Distances ≥5.7 are displayed by the same blue color. Click here for file

Additional file 10 Data of 16S rRNA gene sequences of the different eubacterial phyla. Species nomenclature, genome sizes, 16S rRNA gene copy numbers and accession numbers from the eubacterial taxa used in this study. Click here for file
