# Supplementary material for: Gene copy number variation and its significance in cyanobacterial phylogeny
Source: BMC Microbiol. 2012 Aug 15;12:177. doi: 10.1186/1471-2180-12-177 (PMC3552681; doi:10.1186/1471-2180-12-177)
Supplement: Additional file 1 — Identified gene copies. The sheet contains Information on 41 gene copies and their presence in 22 cyanobacterial species. Amino acid sequences of the coded proteins exhibit 98% similarity within a genome and 50% across species. [file 1471-2180-12-177-S1.pdf]

strain	accession number	gene annotation	gene description	protein accession number	location in the genome
1 1196Cyanothece sp PCC8801 protein	CP001287.1 ACK65303.1	gene=PCC8801 1237	protein=photosystem q(b) protein	protein id=ACK65303.1	location=1288606..1289676
1 1946Cyanothece sp PCC8801 protein	CP001287.1 ACK66053.1	gene=PCC8801 2016	protein=photosystem q(b) protein	protein id=ACK66053.1	location=2090110..2091180
2 1439Synechococcus sp RCC307 protein	CT978603.1 CAK28343.1	gene=psbA	protein=Photosystem II protein D1	protein id=CAK28343.1	location=complement(1279858..1280937)
2 2008Synechococcus sp RCC307 protein	CT978603.1 CAK28912.1	gene=psbA	protein=Photosystem II protein D1	protein id=CAK28912.1	location=complement(1742058..1743137)
2 2182Synechococcus sp RCC307 protein	CT978603.1 CAK29086.1	gene=psbA	protein=Photosystem II protein D1	protein id=CAK29086.1	location=1894037..1895116
5 1594Anabaena variabilis ATCC29413 protein	CP000117.1 ABA21220.1	gene=Ava 1597	protein=Photosystem II reaction centre protein PsbA/D1	protein id=ABA21220.1	location=complement(1971884..1972966)
5 2449Anabaena variabilis ATCC29413 protein	CP000117.1 ABA22075.1	gene=Ava 2460	protein=Photosystem II reaction centre protein PsbA/D1	protein id=ABA22075.1	location=complement(3053603..3054685)
5 3533Anabaena variabilis ATCC29413 protein	CP000117.1 ABA23159.1	gene=Ava 3553	protein=Photosystem II reaction centre protein PsbA/D1	protein id=ABA23159.1	location=complement(4422987..4424069)
6 4108Arthrospira platensis NIES-39 protein	AP011615.1 BAI91950.1	gene=psbAIV	protein=photosystem II reaction center D1 protein	protein id=BAI91950.1	location=complement(4171577..4172656)
6 4113Arthrospira platensis NIES-39 protein	AP011615.1 BAI91955.1	gene=psbAIII	protein=photosystem II reaction center D1 protein	protein id=BAI91955.1	location=complement(4178243..4179322)
6 6481Arthrospira platensis NIES-39 protein	AP011615.1 BAI94323.1	gene=psbAII	protein=photosystem II reaction center D1 protein	protein id=BAI94323.1	location=complement(6629459..6630538)
7 8Synechocystis sp PCC6803 protein	BA000022.2 BAA16586.1	gene=psbA2	protein=photosystem II D1 protein	protein id=BAA16586.1	location=7229..8311
7 1651Synechocystis sp PCC6803 protein	BA000022.2 BAA18230.1	gene=psbA3	protein=photosystem II D1 protein	protein id=BAA18230.1	location=complement(1818915..1819997)
8 779Synechococcus sp JA-3-3Ab protein	BA000045.2 BAC88720.1	gene=psbA	protein=photosystem II protein D1	protein id=BAC88720.1	location=832184..833266
8 2322Synechococcus sp JA-3-3Ab protein	BA000045.2 BAC90263.1	gene=psbA	protein=photosystem II protein D1	protein id=BAC90263.1	location=2486614..2487696
8 3143Synechococcus sp JA-3-3Ab protein	BA000045.2 BAC91085.1	gene=psbA	protein=photosystem II protein D1	protein id=BAC91085.1	location=complement(3348383..3349465)
9 165Cyanothece sp PCC7424 protein	CP001291.1 ACK68639.1	gene=PCC7424 0169	protein=photosystem q(b) protein	protein id=ACK68639.1	location=186554..187624
9 1028Cyanothece sp PCC7424 protein	CP001291.1 ACK69503.1	gene=PCC7424 1049	protein=photosystem q(b) protein	protein id=ACK69503.1	location=1150654..1151724
9 2240Cyanothece sp PCC7424 protein	CP001291.1 ACK70715.1	gene=PCC7424 2293	protein=photosystem q(b) protein	protein id=ACK70715.1	location=complement(2562885..2563955)
10 405Prochlorococcus marinus str MIT9303 protein	CP000554.1 ABM77161.1	gene=psbA	protein=Photosystem II PsbA protein (D1)	protein id=ABM77161.1	location=406385..407461
10 1853Prochlorococcus marinus str MIT9303 protein	CP000554.1 ABM78610.1	gene=P9303 18681	protein=Hypothetical protein	protein id=ABM78610.1	location=1629607..1630683
12 3568Nostoc sp PCC7120 protein	BA000019.2 BAB75271.1	gene=psbAIV	protein=photosystem II protein D1	protein id=BAB75271.1	location=complement(4315437..4316519)
12 3723Nostoc sp PCC7120 protein	BA000019.2 BAB75426.1	gene=psbAII	protein=photosystem II protein D1	protein id=BAB75426.1	location=4499998..4501080
12 4587Nostoc sp PCC7120 protein	BA000019.2 BAB76291.1	gene=psbAIII	protein=photosystem II protein D1	protein id=BAB76291.1	location=5489831..5490913
14 1021Microcystis aeruginosa NIES-843 protein	AP009552.1 BAG00844.1	gene=psbA1	protein=photosystem II D1 protein	protein id=BAG00844.1	location=complement(877533..878615)
14 1037Microcystis aeruginosa NIES-843 protein	AP009552.1 BAG00860.1	gene=psbA2	protein=photosystem II D1 protein	protein id=BAG00860.1	location=891585..892667
14 1050Microcystis aeruginosa NIES-843 protein	AP009552.1 BAG00873.1	gene=psbA3	protein=photosystem II D1 protein	protein id=BAG00873.1	location=900555..901637
14 1079Microcystis aeruginosa NIES-843 protein	AP009552.1 BAG00902.1	gene=psbA4	protein=photosystem II D1 protein	protein id=BAG00902.1	location=925990..927072
14 5813Microcystis aeruginosa NIES-843 protein	AP009552.1 BAG05636.1	gene=psbA5	protein=photosystem II D1 protein	protein id=BAG05636.1	location=5351373..5352455
15 584Nostoc azollae 0708 protein	CP002059.1 ADI63253.1	gene=Aazo 0830	protein=photosystem q(b) protein	protein id=ADI63253.1	location=879395..880477
15 1149Nostoc azollae 0708 protein	CP002059.1 ADI63818.1	gene=Aazo 1652	protein=photosystem q(b) protein	protein id=ADI63818.1	location=1720331..1721413
16 2089Acharyochloris marina MBIC11017 protein	CP000828.1 ABW27180.1	gene=psbA	protein=photosystem II D1 protein PsbA	protein id=ABW27180.1	location=complement(2166714..2167796)
16 2799Acharyochloris marina MBIC11017 protein	CP000828.1 ABW27888.1	gene=psbA	protein=photosystem II D1 protein PsbA	protein id=ABW27888.1	location=complement(2928273..2929355)
17 2057Nostoc punctiforme PCC73102 protein	CP001037.1 ACC80861.1	gene=Npun R2273	protein=photosystem q(b) protein	protein id=ACC80861.1	location=complement(2808705..2809787)
17 3144Nostoc punctiforme PCC73102 protein	CP001037.1 ACC81948.1	gene=Npun F3544	protein=photosystem q(b) protein	protein id=ACC81948.1	location=4451534..4452616
17 4716Nostoc punctiforme PCC73102 protein	CP001037.1 ACC83520.1	gene=Npun R5188	protein=photosystem q(b) protein	protein id=ACC83520.1	location=complement(6424030..6425112)

18 166Synechococcus elongatus PCC6301 protein	AP008231.1 BAD78356.1	gene=psbAII	protein=photosystem II D1 protein	protein id=BAD78356.1	location=185180..186262
18 647Synechococcus elongatus PCC6301 protein	AP008231.1 BAD78837.1	gene=psbAIII	protein=photosystem II D1 protein	protein id=BAD78837.1	location=complement(716582..717664)
19 779Gloeobacter violaceus PCC7421 protein	BA000045.2 BAC88720.1	gene=psbA	protein=photosystem II protein D1	protein id=BAC88720.1	location=832184..833266
19 2322Gloeobacter violaceus PCC7421 protein	BA000045.2 BAC90263.1	gene=psbA	protein=photosystem II protein D1	protein id=BAC90263.1	location=2486614..2487696
19 3143Gloeobacter violaceus PCC7421 protein	BA000045.2 BAC91085.1	gene=psbA	protein=photosystem II protein D1	protein id=BAC91085.1	location=complement(3348383..3349465)
20 143Trichodesmium erythraeum IMS101 protein	CP000393.1 ABG49673.1	gene=Tery 0182	protein=photosystem q(b) protein	protein id=ABG49673.1	location=269811..270872
20 144Trichodesmium erythraeum IMS101 protein	CP000393.1 ABG49674.1	gene=Tery 0183	protein=photosystem q(b) protein	protein id=ABG49674.1	location=271328..272389
20 4186Trichodesmium erythraeum IMS101 protein	CP000393.1 ABG53717.1	gene=Tery 4763	protein=photosystem q(b) protein	protein id=ABG53717.1	location=complement(7315951..7317012)
21 366Synechococcus sp PCC7803 protein	CT971583.1 CAK22792.1	gene=psbA	protein=Photosystem II protein D1	protein id=CAK22792.1	location=complement(384540..385619)
21 790Synechococcus sp PCC7803 protein	CT971583.1 CAK23216.1	gene=psbA	protein=Photosystem II protein D1	protein id=CAK23216.1	location=776911..777990
21 2084Synechococcus sp PCC7803 protein	CT971583.1 CAK24510.1	gene=psbA	protein=Photosystem II protein D1	protein id=CAK24510.1	location=complement(1914146..1915225)
22 153Synechococcus sp PCC7002 protein	CP000951.1 ACA98171.1	gene=psbA-II	protein=photosystem II D1 subunit PsbA-II (Qb protein)	protein id=ACA98171.1	location=complement(161801..162847)
22 1391Synechococcus sp PCC7002 protein	CP000951.1 ACA99409.1	gene=psbA	protein=photosystem q(b) protein	protein id=ACA99409.1	location=1489906..1490988
1 56Cyanothece sp PCC8801 protein	CP001287.1 ACK64163.1	gene=PCC8801 0056	protein=photosystem II D2 protein (photosystem q(a) protein)	protein id=ACK64163.1	location=complement(58944..60002)
1 1891Cyanothece sp PCC8801 protein	CP001287.1 ACK65998.1	gene=PCC8801 1960	protein=photosystem II D2 protein (photosystem q(a) protein)	protein id=ACK65998.1	location=2036321..2037379
2 258Synechococcus sp RCC307 protein	CT978603.1 CAK27162.1	gene=psbD	protein=Photosystem II D2 protein	protein id=CAK27162.1	location=256888..257946
2 1694Synechococcus sp RCC307 protein	CT978603.1 CAK28598.1	gene=psbD	protein=Photosystem II D2 protein	protein id=CAK28598.1	location=1479498..1480556
3 454Thermosynechococcus elongatus BP-1 protein	BA000039.2 BAC08007.1	gene=psbD2	protein=photosystem II reaction center D2 protein	protein id=BAC08007.1	location=456131..457189
3 1628Thermosynechococcus elongatus BP-1 protein	BA000039.2 BAC09182.1	gene=psbD1	protein=photosystem II reaction center D2 protein	protein id=BAC09182.1	location=1702500..1703558
5 1240Anabaena variabilis ATCC29413 protein	CP000117.1 ABA20866.1	gene=Ava 1242	protein=Photosystem II reaction centre protein PsbD/D2	protein id=ABA20866.1	location=1529297..1530352
5 2501Anabaena variabilis ATCC29413 protein	CP000117.1 ABA22127.1	gene=Ava 2512	protein=Photosystem II reaction centre protein PsbD/D2	protein id=ABA22127.1	location=complement(3106302..3107357)
6 1954Arthrospira platensis NIES-39 protein	AP011615.1 BAI89796.1	gene=psbDII	protein=photosystem II reaction center D2 protein	protein id=BAI89796.1	location=1968930..1969988
6 3148Arthrospira platensis NIES-39 protein	AP011615.1 BAI90990.1	gene=psbD1	protein=photosystem II reaction center D2 protein	protein id=BAI90990.1	location=3179356..3180414
7 1221Synechocystis sp PCC6803 protein	BA000022.2 BAA17800.1	gene=psbD	protein=photosystem II D2 protein	protein id=BAA17800.1	location=complement(1348840..1349898)
7 2881Synechocystis sp PCC6803 protein	BA000022.2 BAA10851.1	gene=psbD2	protein=photosystem II D2 protein	protein id=BAA10851.1	location=3229780..3230838
9 566Cyanothece sp PCC7424 protein	CP001291.1 ACK69040.1	gene=PCC7424 0579	protein=photosystem II D2 protein (photosystem q(a) protein)	protein id=ACK69040.1	location=complement(636371..637429)
9 2902Cyanothece sp PCC7424 protein	CP001291.1 ACK71377.1	gene=PCC7424 2974	protein=photosystem II D2 protein (photosystem q(a) protein)	protein id=ACK71377.1	location=complement(3317859..3318917)
12 4285Nostoc sp PCC7120 protein	BA000019.2 BAB75989.1	gene=psbD	protein=photosystem II protein D2	protein id=BAB75989.1	location=5143731..5144786
12 4543Nostoc sp PCC7120 protein	BA000019.2 BAB76247.1	gene=psbD	protein=photosystem II protein D2	protein id=BAB76247.1	location=5441150..5442205
14 1797Microcystis aeruginosa NIES-843 protein	AP009552.1 BAG01620.1	gene=psbD2	protein=photosystem II reaction center D2 protein	protein id=BAG01620.1	location=1613898..1614953
14 4112Microcystis aeruginosa NIES-843 protein	AP009552.1 BAG03938.1	gene=psbD1	protein=photosystem II reaction center D2 protein	protein id=BAG03938.1	location=complement(3780970..3782025)
15 747Nostoc azollae 0708 protein	CP002059.1 ADI63416.1	gene=Aazo 1059	protein=photosystem II D2 protein (photosystem q(a) protein)	protein id=ADI63416.1	location=1131903..1132958
15 2645Nostoc azollae 0708 protein	CP002059.1 ADI65314.1	gene=Aazo 3778	protein=photosystem II D2 protein (photosystem q(a) protein)	protein id=ADI65314.1	location=3834890..3835945
16 1031Acharyochloris marina MBIC11017 protein	CP000828.1 ABW26122.1	gene=psbD	protein=photosystem II D2 protein PsbD	protein id=ABW26122.1	location=1050536..1051591
16 3977Acharyochloris marina MBIC11017 protein	CP000828.1 ABW29065.1	gene=psbD	protein=photosystem II D2 protein PsbD	protein id=ABW29065.1	location=complement(4131304..4132359)
17 3223Nostoc punctiforme PCC73102 protein	CP001037.1 ACC82027.1	gene=Npun R3637	protein=photosystem II D2 protein (photosystem q(a) protein)	protein id=ACC82027.1	location=complement(4583337..4584392)
17 4110Nostoc punctiforme PCC73102 protein	CP001037.1 ACC82914.1	gene=Npun F4553	protein=photosystem II D2 protein (photosystem q(a) protein)	protein id=ACC82914.1	location=5658199..5659254
18 873Synechococcus elongatus PCC6301 protein	AP008231.1 BAD79063.1	gene=psbDI	protein=photosystem II reaction center D2 protein	protein id=BAD79063.1	location=complement(969581..970639)
18 2447Synechococcus elongatus PCC6301 protein	AP008231.1 BAD80638.1	gene=psbDII	protein=photosystem II reaction center D2 protein	protein id=BAD80638.1	location=2610565..2611623
21 1647Synechococcus sp PCC7803 protein	CT971583.1 CAK24073.1	gene=psbD	protein=Photosystem II D2 protein	protein id=CAK24073.1	location=1513257..1514312
21 2239Synechococcus sp PCC7803 protein	CT971583.1 CAK24665.1	gene=psbD	protein=Photosystem II D2 protein	protein id=CAK24665.1	location=complement(2064063..2065118)
22 1533Synechococcus sp PCC7002 protein	CP000951.1 ACA99551.1	gene=psbD	protein=photosystem II D2 protein (photosystem q(a) protein)	protein id=ACA99551.1	location=complement(1646526..1647584)
22 2161Synechococcus sp PCC7002 protein	CP000951.1 ACB00180.1	gene=psbD	protein=photosystem II D2 protein	protein id=ACB00180.1	location=2288194..2289252

strain	accession number	gene annotation	gene description	protein accession number	location in the genome
1 3242Cyanothece sp PCC8801 protein	CP001287.1 ACK67350.1	gene=PCC8801 3382	protein=transposase IS4 family protein	protein id=ACK67350.1	location=complement(3528365..3529384)
1 3688Cyanothece sp PCC8801 protein	CP001287.1 ACK67796.1	gene=PCC8801 3846	protein=transposase IS4 family protein	protein id=ACK67796.1	location=complement(4025990..4027009)
5 261Anabaena variabilis ATCC29413 protein	CP000117.1 ABA19887.1	gene=Ava 0261	protein=transposase, IS4 family	protein id=ABA19887.1	location=334202..335224
5 3135Anabaena variabilis ATCC29413 protein	CP000117.1 ABA22761.1	gene=Ava 3153	protein=transposase, IS4 family	protein id=ABA22761.1	location=3914670..3915692
7 1078Synechocystis sp PCC6803 protein	BA000022.2 BAA17657.1	gene=sll1780	protein=transposase	protein id=BAA17657.1	location=complement(1200376..1201392)
7 1576Synechocystis sp PCC6803 protein	BA000022.2 BAA18155.1	gene=sll1255	protein=transposase	protein id=BAA18155.1	location=complement(1729012..1730028)
7 1793Synechocystis sp PCC6803 protein	BA000022.2 BAA18372.1	gene=sll1560	protein=transposase	protein id=BAA18372.1	location=complement(1970952..1971968)
7 1868Synechocystis sp PCC6803 protein	BA000022.2 BAA18447.1	gene=slr1635	protein=transposase	protein id=BAA18447.1	location=2048497..2049513
7 2098Synechocystis sp PCC6803 protein	BA000022.2 BAA10067.1	gene=slr0180	protein=transposase	protein id=BAA10067.1	location=2327013..2328029
7 3020Synechocystis sp PCC6803 protein	BA000022.2 BAA18731.1	gene=sll1474	protein=transposase	protein id=BAA18731.1	location=complement(3400402..3401418)
8 761Synechococcus sp JA-3-3Ab protein	BA000045.2 BAC88702.1	gene=glr0761	protein=glr0761	protein id=BAC88702.1	location=813826..814881
8 1195Synechococcus sp JA-3-3Ab protein	BA000045.2 BAC89136.1	gene=gll1195	protein=gll1195	protein id=BAC89136.1	location=complement(1271101..1272156)
17 1266Nostoc punctiforme PCC73102 protein	CP001037.1 ACC80069.1	gene=Npun F1359	protein=transposase, IS4 family protein	protein id=ACC80069.1	location=1653292..1654314
17 1374Nostoc punctiforme PCC73102 protein	CP001037.1 ACC80177.1	gene=Npun F1481	protein=transposase, IS4 family protein	protein id=ACC80177.1	location=1818320..1819342
17 2675Nostoc punctiforme PCC73102 protein	CP001037.1 ACC81479.1	gene=Npun F2948	protein=transposase, IS4 family protein	protein id=ACC81479.1	location=3648697..3649719
17 3118Nostoc punctiforme PCC73102 protein	CP001037.1 ACC81922.1	gene=Npun F3516	protein=transposase, IS4 family protein	protein id=ACC81922.1	location=4420430..4421452
17 3218Nostoc punctiforme PCC73102 protein	CP001037.1 ACC82022.1	gene=Npun F3628	protein=transposase, IS4 family protein	protein id=ACC82022.1	location=4572908..4573930
19 761Gloeobacter violaceus PCC7421 protein	BA000045.2 BAC88702.1	gene=glr0761	protein=glr0761	protein id=BAC88702.1	location=813826..814881
19 1195Gloeobacter violaceus PCC7421 protein	BA000045.2 BAC89136.1	gene=gll1195	protein=gll1195	protein id=BAC89136.1	location=complement(1271101..1272156)
8 872Synechococcus sp JA-3-3Ab protein	BA000045.2 BAC88813.1	gene=glr0872	protein=glr0872	protein id=BAC88813.1	location=919913..920968
8 1093Synechococcus sp JA-3-3Ab protein	BA000045.2 BAC89034.1	gene=gll1093	protein=gll1093	protein id=BAC89034.1	location=complement(1171434..1172489)
8 2126Synechococcus sp JA-3-3Ab protein	BA000045.2 BAC90067.1	gene=gll2126	protein=gll2126	protein id=BAC90067.1	location=complement(2280085..2281140)
19 872Gloeobacter violaceus PCC7421 protein	BA000045.2 BAC88813.1	gene=glr0872	protein=glr0872	protein id=BAC88813.1	location=919913..920968
19 1093Gloeobacter violaceus PCC7421 protein	BA000045.2 BAC89034.1	gene=gll1093	protein=gll1093	protein id=BAC89034.1	location=complement(1171434..1172489)
19 2126Gloeobacter violaceus PCC7421 protein	BA000045.2 BAC90067.1	gene=gll2126	protein=gll2126	protein id=BAC90067.1	location=complement(2280085..2281140)
8 209Synechococcus sp JA-3-3Ab protein	BA000045.2 BAC88150.1	gene=gll0209	protein=gll0209	protein id=BAC88150.1	location=complement(202099..203154)
8 1105Synechococcus sp JA-3-3Ab protein	BA000045.2 BAC89046.1	gene=gll1105	protein=gll1105	protein id=BAC89046.1	location=complement(1185264..1186319)
19 209Gloeobacter violaceus PCC7421 protein	BA000045.2 BAC88150.1	gene=gll0209	protein=gll0209	protein id=BAC88150.1	location=complement(202099..203154)
19 1105Gloeobacter violaceus PCC7421 protein	BA000045.2 BAC89046.1	gene=gll1105	protein=gll1105	protein id=BAC89046.1	location=complement(1185264..1186319)
6 3383Arthrospira platensis NIES-39 protein	AP011615.1 BAI91225.1	gene=NIES39 J01730	protein=putative transposase	protein id=BAI91225.1	location=3451691..3452098
6 3707Arthrospira platensis NIES-39 protein	AP011615.1 BAI91549.1	gene=NIES39 J05030	protein=putative transposase	protein id=BAI91549.1	location=3760401..3760823
6 4464Arthrospira platensis NIES-39 protein	AP011615.1 BAI92306.1	gene=NIES39 L01450	protein=putative transposase	protein id=BAI92306.1	location=complement(4567432..4567854)
8 1611Synechococcus sp JA-3-3Ab protein	BA000045.2 BAC89552.1	gene=gll1611	protein=gll1611	protein id=BAC89552.1	location=complement(1728502..1728960)
8 1727Synechococcus sp JA-3-3Ab protein	BA000045.2 BAC89668.1	gene=glr1727	protein=glr1727	protein id=BAC89668.1	location=1833160..1833618
8 4074Synechococcus sp JA-3-3Ab protein	BA000045.2 BAC92017.1	gene=glr4076	protein=glr4076	protein id=BAC92017.1	location=4271569..4272027

16 1072Acharyochloris marina MBIC11017 protein	CP000828.1 ABW26163.1	gene=AM1 1124	protein=transposase, putative	protein id=ABW26163.1	location=1099836..1100309
16 1257Acharyochloris marina MBIC11017 protein	CP000828.1 ABW26348.1	gene=AM1 1314	protein=transposase, putative	protein id=ABW26348.1	location=1300852..1301325
16 3820Acharyochloris marina MBIC11017 protein	CP000828.1 ABW28908.1	gene=AM1 3923	protein=conserved hypothetical protein	protein id=ABW28908.1	location=3974665..3975138
19 1611Gloeobacter violaceus PCC7421 protein	BA000045.2 BAC89552.1	gene=gll1611	protein=gll1611	protein id=BAC89552.1	location=complement(1728502..1728960)
19 1727Gloeobacter violaceus PCC7421 protein	BA000045.2 BAC89668.1	gene=glr1727	protein=glr1727	protein id=BAC89668.1	location=1833160..1833618
19 4074Gloeobacter violaceus PCC7421 protein	BA000045.2 BAC92017.1	gene=glr4076	protein=glr4076	protein id=BAC92017.1	location=4271569..4272027
20 1086Trichodesmium erythraeum IMS101 protein	CP000393.1 ABG50616.1	gene=Tery 1283	protein=hypothetical protein	protein id=ABG50616.1	location=1963943..1964374
20 1992Trichodesmium erythraeum IMS101 protein	CP000393.1 ABG51522.1	gene=Tery 2298	protein=hypothetical protein	protein id=ABG51522.1	location=complement(3567237..3567647)
20 2498Trichodesmium erythraeum IMS101 protein	CP000393.1 ABG52028.1	gene=Tery 2854	protein=hypothetical protein	protein id=ABG52028.1	location=complement(4445965..4446375)
20 2672Trichodesmium erythraeum IMS101 protein	CP000393.1 ABG52203.1	gene=Tery 3055	protein=hypothetical protein	protein id=ABG52203.1	location=complement(4715752..4716162)
16 452Acharyochloris marina MBIC11017 protein	CP000828.1 ABW25543.1	gene=AM1 0490	protein=transposase, putative	protein id=ABW25543.1	location=469494..469901
16 462Acharyochloris marina MBIC11017 protein	CP000828.1 ABW25553.1	gene=AM1 0500	protein=transposase, putative	protein id=ABW25553.1	location=complement(477089..477496)
16 2018Acharyochloris marina MBIC11017 protein	CP000828.1 ABW27109.1	gene=AM1 2094	protein=conserved hypothetical protein	protein id=ABW27109.1	location=2086211..2086618
5 798Anabaena variabilis ATCC29413 protein	CP000117.1 ABA20424.1	gene=Ava 0800	protein=transposase	protein id=ABA20424.1	location=981653..982036
5 1016Anabaena variabilis ATCC29413 protein	CP000117.1 ABA20642.1	gene=Ava 1018	protein=transposase	protein id=ABA20642.1	location=1228979..1229362
5 1362Anabaena variabilis ATCC29413 protein	CP000117.1 ABA20988.1	gene=Ava 1364	protein=transposase	protein id=ABA20988.1	location=complement(1680843..1681226)
5 2629Anabaena variabilis ATCC29413 protein	CP000117.1 ABA22255.1	gene=Ava 2641	protein=transposase	protein id=ABA22255.1	location=3269211..3269594
5 3298Anabaena variabilis ATCC29413 protein	CP000117.1 ABA22924.1	gene=Ava 3317	protein=transposase	protein id=ABA22924.1	location=4141228..4141611
5 3779Anabaena variabilis ATCC29413 protein	CP000117.1 ABA23405.1	gene=Ava 3800	protein=transposase	protein id=ABA23405.1	location=4735420..4735803
7 784Synechocystis sp PCC6803 protein	BA000022.2 BAA17363.1	gene=sll1791	protein=transposase	protein id=BAA17363.1	location=complement(852895..853281)
7 2741Synechocystis sp PCC6803 protein	BA000022.2 BAA10711.1	gene=slr0799	protein=transposase	protein id=BAA10711.1	location=3066429..3066752
12 16Nostoc sp PCC7120 protein	BA000019.2 BAB77540.1	gene=all0016	protein=transposase	protein id=BAB77540.1	location=complement(13138..13521)
12 2689Nostoc sp PCC7120 protein	BA000019.2 BAB74392.1	gene=all2693	protein=transposase	protein id=BAB74392.1	location=complement(3284646..3285029)
12 3606Nostoc sp PCC7120 protein	BA000019.2 BAB75309.1	gene=alr3610	protein=transposase	protein id=BAB75309.1	location=4361124..4361507
12 4395Nostoc sp PCC7120 protein	BA000019.2 BAB76099.1	gene=all4400	protein=transposase	protein id=BAB76099.1	location=complement(5276131..5276514)
12 4433Nostoc sp PCC7120 protein	BA000019.2 BAB76137.1	gene=alr4438	protein=transposase	protein id=BAB76137.1	location=5322370..5322753
12 4812Nostoc sp PCC7120 protein	BA000019.2 BAB76516.1	gene=all4817	protein=transposase	protein id=BAB76516.1	location=complement(5735861..5736244)
12 5152Nostoc sp PCC7120 protein	BA000019.2 BAB76856.1	gene=alr5157	protein=transposase	protein id=BAB76856.1	location=6156759..6157142
15 3396Nostoc azollae 0708 protein	CP002059.1 ADI66065.1	gene=Aazo 4932	protein=transposase	protein id=ADI66065.1	location=complement(5047600..5047983)
15 3550Nostoc azollae 0708 protein	CP002059.1 ADI66219.1	gene=Aazo 5164	protein=transposase	protein id=ADI66219.1	location=5290637..5291020
15 322Nostoc azollae 0708 protein	CP002059.1 ADI62991.1	gene=Aazo 0453	protein=transposase	protein id=ADI62991.1	location=468637..469020
15 3126Nostoc azollae 0708 protein	CP002059.1 ADI65795.1	gene=Aazo 4525	protein=transposase	protein id=ADI65795.1	location=4617611..4617994
6 4008Arthrospira platensis NIES-39 protein	AP011615.1 BAI91850.1	gene=NIES39 K02030	protein=putative transposase	protein id=BAI91850.1	location=4067817..4068176
6 4011Arthrospira platensis NIES-39 protein	AP011615.1 BAI91853.1	gene=NIES39 K02060	protein=putative transposase	protein id=BAI91853.1	location=complement(4069017..4069376)
8 1612Synechococcus sp JA-3-3Ab protein	BA000045.2 BAC89553.1	gene=gll1612	protein=gll1612	protein id=BAC89553.1	location=complement(1728993..1729379)
8 1726Synechococcus sp JA-3-3Ab protein	BA000045.2 BAC89667.1	gene=glr1726	protein=glr1726	protein id=BAC89667.1	location=1832741..1833127
8 4073Synechococcus sp JA-3-3Ab protein	BA000045.2 BAC92016.1	gene=glr4075	protein=glr4075	protein id=BAC92016.1	location=4271150..4271536
19 1612Gloeobacter violaceus PCC7421 protein	BA000045.2 BAC89553.1	gene=gll1612	protein=gll1612	protein id=BAC89553.1	location=complement(1728993..1729379)
19 1726Gloeobacter violaceus PCC7421 protein	BA000045.2 BAC89667.1	gene=glr1726	protein=glr1726	protein id=BAC89667.1	location=1832741..1833127
19 4073Gloeobacter violaceus PCC7421 protein	BA000045.2 BAC92016.1	gene=glr4075	protein=glr4075	protein id=BAC92016.1	location=4271150..4271536

6 2123Arthrospira platensis NIES-39 protein	AP011615.1 BAI89965.1	gene=NIES39 D05480	protein=putative transposase	protein id=BAI89965.1	location=2128937..2129296
6 3647Arthrospira platensis NIES-39 protein	AP011615.1 BAI91489.1	gene=NIES39 J04420	protein=putative transposase	protein id=BAI91489.1	location=complement(3710514..3710873)
20 1085Trichodesmium erythraeum IMS101 protein	CP000393.1 ABG50615.1	gene=Tery 1282	protein=putative transposase	protein id=ABG50615.1	location=1963528..1963887
20 1993Trichodesmium erythraeum IMS101 protein	CP000393.1 ABG51523.1	gene=Tery 2299	protein=putative transposase	protein id=ABG51523.1	location=complement(3567724..3568083)
20 2499Trichodesmium erythraeum IMS101 protein	CP000393.1 ABG52029.1	gene=Tery 2855	protein=putative transposase	protein id=ABG52029.1	location=complement(4446452..4446811)
20 2673Trichodesmium erythraeum IMS101 protein	CP000393.1 ABG52204.1	gene=Tery 3056	protein=putative transposase	protein id=ABG52204.1	location=complement(4716239..4716598)
1 1353Cyanothece sp PCC8801 protein	CP001287.1 ACK65460.1	gene=PCC8801 1400	protein=RNA-directed DNA polymerase (Reverse transcriptase)	protein id=ACK65460.1	location=1464222..1466045
1 1354Cyanothece sp PCC8801 protein	CP001287.1 ACK65461.1	gene=PCC8801 1402	protein=RNA-directed DNA polymerase (Reverse transcriptase)	protein id=ACK65461.1	location=1466831..1468654
1 2846Cyanothece sp PCC8801 protein	CP001287.1 ACK66954.1	gene=PCC8801 2962	protein=RNA-directed DNA polymerase (Reverse transcriptase)	protein id=ACK66954.1	location=3059473..3061296
1 2847Cyanothece sp PCC8801 protein	CP001287.1 ACK66955.1	gene=PCC8801 2964	protein=RNA-directed DNA polymerase (Reverse transcriptase)	protein id=ACK66955.1	location=3062082..3063905
6 452Arthrospira platensis NIES-39 protein	AP011615.1 BAI88294.1	gene=NIES39 A04560	protein=reverse transcriptase homolog	protein id=BAI88294.1	location=455495..457261
6 998Arthrospira platensis NIES-39 protein	AP011615.1 BAI88840.1	gene=NIES39 B00830	protein=reverse transcriptase homolog	protein id=BAI88840.1	location=985697..987463
6 1914Arthrospira platensis NIES-39 protein	AP011615.1 BAI89756.1	gene=NIES39 D03370	protein=reverse transcriptase homolog	protein id=BAI89756.1	location=complement(1937168..1938934)
6 4356Arthrospira platensis NIES-39 protein	AP011615.1 BAI92198.1	gene=NIES39 L00370	protein=reverse transcriptase homolog	protein id=BAI92198.1	location=complement(4471823..4473589)
6 5007Arthrospira platensis NIES-39 protein	AP011615.1 BAI92849.1	gene=NIES39 M00110	protein=reverse transcriptase homolog	protein id=BAI92849.1	location=complement(5088414..5090180)
6 6149Arthrospira platensis NIES-39 protein	AP011615.1 BAI93991.1	gene=NIES39 P00110	protein=reverse transcriptase homolog	protein id=BAI93991.1	location=6262865..6264631
9 949Cyanothece sp PCC7424 protein	CP001291.1 ACK69423.1	gene=PCC7424 0968	protein=RNA-directed DNA polymerase (Reverse transcriptase)	protein id=ACK69423.1	location=complement(1073514..1075460)
9 3260Cyanothece sp PCC7424 protein	CP001291.1 ACK71735.1	gene=PCC7424 3336	protein=RNA-directed DNA polymerase (Reverse transcriptase)	protein id=ACK71735.1	location=3733467..3735413
14 585Microcystis aeruginosa NIES-843 protein	AP009552.1 BAG00407.1	gene=MAE 05850	protein=putative group II intron/maturase	protein id=BAG00407.1	location=511461..513305
14 1995Microcystis aeruginosa NIES-843 protein	AP009552.1 BAG01818.1	gene=MAE 19960	protein=RNA-directed DNA polymerase	protein id=BAG01818.1	location=complement(1788827..1790671)
14 6204Microcystis aeruginosa NIES-843 protein	AP009552.1 BAG06027.1	gene=MAE 62050	protein=probable reverse transcriptase	protein id=BAG06027.1	location=5747312..5749156
20 196Trichodesmium erythraeum IMS101 protein	CP000393.1 ABG49726.1	gene=Tery 0239	protein=RNA-directed DNA polymerase	protein id=ABG49726.1	location=373560..375467
20 2880Trichodesmium erythraeum IMS101 protein	CP000393.1 ABG52411.1	gene=Tery 3305	protein=RNA-directed DNA polymerase	protein id=ABG52411.1	location=5068307..5070214
5 78Anabaena variabilis ATCC29413 protein	CP000117.1 ABA19704.1	gene=Ava 0078	protein=Gas vesicle protein GVPa	protein id=ABA19704.1	location=complement(100886..101101)
5 79Anabaena variabilis ATCC29413 protein	CP000117.1 ABA19705.1	gene=Ava 0079	protein=Gas vesicle protein GVPa	protein id=ABA19705.1	location=complement(101206..101421)
12 2250Nostoc sp PCC7120 protein	BA000019.2 BAB73952.1	gene=gvpB	protein=gas vesicle protein	protein id=BAB73952.1	location=complement(2706132..2706347)
12 2251Nostoc sp PCC7120 protein	BA000019.2 BAB73953.1	gene=gvpA	protein=gas vesicle protein	protein id=BAB73953.1	location=complement(2706452..2706667)
14 3754Microcystis aeruginosa NIES-843 protein	AP009552.1 BAG03580.1	gene=gvpAI	protein=gas vesicle protein GvpAI	protein id=BAG03580.1	location=3399385..3399600
14 3755Microcystis aeruginosa NIES-843 protein	AP009552.1 BAG03581.1	gene=gvpAII	protein=gas vesicle protein GvpAII	protein id=BAG03581.1	location=3399955..3400170
14 3756Microcystis aeruginosa NIES-843 protein	AP009552.1 BAG03582.1	gene=gvpAIII	protein=gas vesicle protein GvpAIII	protein id=BAG03582.1	location=3400500..3400715
5 1438Anabaena variabilis ATCC29413 protein	CP000117.1 ABA21064.1	gene=Ava 1441	protein=transposase, IS4 family	protein id=ABA21064.1	location=1783089..1783529
5 2475Anabaena variabilis ATCC29413 protein	CP000117.1 ABA22101.1	gene=Ava 2486	protein=transposase, IS4 family	protein id=ABA22101.1	location=complement(3082253..3082642)
12 15Nostoc sp PCC7120 protein	BA000019.2 BAB77539.1	gene=all0015	protein=transposase	protein id=BAB77539.1	location=complement(12728..13168)
12 3607Nostoc sp PCC7120 protein	BA000019.2 BAB75310.1	gene=alr3611	protein=transposase	protein id=BAB75310.1	location=4361477..4361917
12 4394Nostoc sp PCC7120 protein	BA000019.2 BAB76098.1	gene=all4399	protein=transposase	protein id=BAB76098.1	location=complement(5275721..5276161)
12 4434Nostoc sp PCC7120 protein	BA000019.2 BAB76138.1	gene=alr4439	protein=transposase	protein id=BAB76138.1	location=5322723..5323163
12 4811Nostoc sp PCC7120 protein	BA000019.2 BAB76515.1	gene=all4816	protein=transposase	protein id=BAB76515.1	location=complement(5735451..5735891)
12 5153Nostoc sp PCC7120 protein	BA000019.2 BAB76857.1	gene=alr5158	protein=transposase	protein id=BAB76857.1	location=6157112..6157552
15 1014Nostoc azollae 0708 protein	CP002059.1 ADI63683.1	gene=Aazo 1465	protein=transposase IS4 family protein	protein id=ADI63683.1	location=complement(1532502..1532957)
15 1308Nostoc azollae 0708 protein	CP002059.1 ADI63977.1	gene=Aazo 1882	protein=transposase IS4 family protein	protein id=ADI63977.1	location=complement(1955990..1956445)
15 1612Nostoc azollae 0708 protein	CP002059.1 ADI64281.1	gene=Aazo 2318	protein=transposase IS4 family protein	protein id=ADI64281.1	location=complement(2406642..2407097)

8 1184Synechococcus sp JA-3-3Ab protein	BA000045.2 BAC89125.1	gene=cpcB	protein=phycocyanin beta chain	protein id=BAC89125.1	location=1263334..1263852
8 3216Synechococcus sp JA-3-3Ab protein	BA000045.2 BAC91158.1	gene=cpcB	protein=phycocyanin beta chain	protein id=BAC91158.1	location=3424755..3425273
18 496Synechococcus elongatus PCC6301 protein	AP008231.1 BAD78686.1	gene=cpcB	protein=phycocyanin beta subunit	protein id=BAD78686.1	location=complement(556210..556731)
18 501Synechococcus elongatus PCC6301 protein	AP008231.1 BAD78691.1	gene=cpcB	protein=phycocyanin beta subunit	protein id=BAD78691.1	location=complement(559965..560486)
19 1184Gloeobacter violaceus PCC7421 protein	BA000045.2 BAC89125.1	gene=cpcB	protein=phycocyanin beta chain	protein id=BAC89125.1	location=1263334..1263852
19 3216Gloeobacter violaceus PCC7421 protein	BA000045.2 BAC91158.1	gene=cpcB	protein=phycocyanin beta chain	protein id=BAC91158.1	location=3424755..3425273
8 1185Synechococcus sp JA-3-3Ab protein	BA000045.2 BAC89126.1	gene=cpcA	protein=phycocyanin alpha chain	protein id=BAC89126.1	location=1263891..1264379
8 3217Synechococcus sp JA-3-3Ab protein	BA000045.2 BAC91159.1	gene=cpcA	protein=phycocyanin alpha chain	protein id=BAC91159.1	location=3425312..3425800
18 495Synechococcus elongatus PCC6301 protein	AP008231.1 BAD78685.1	gene=cpcA	protein=phycocyanin alpha subunit	protein id=BAD78685.1	location=complement(555667..556158)
18 500Synechococcus elongatus PCC6301 protein	AP008231.1 BAD78690.1	gene=cpcA	protein=phycocyanin alpha subunit	protein id=BAD78690.1	location=complement(559422..559913)
19 1185Gloeobacter violaceus PCC7421 protein	BA000045.2 BAC89126.1	gene=cpcA	protein=phycocyanin alpha chain	protein id=BAC89126.1	location=1263891..1264379
19 3217Gloeobacter violaceus PCC7421 protein	BA000045.2 BAC91159.1	gene=cpcA	protein=phycocyanin alpha chain	protein id=BAC91159.1	location=3425312..3425800
8 146Synechococcus sp JA-3-3Ab protein	BA000045.2 BAC88087.1	gene=glr0146	protein=glr0146	protein id=BAC88087.1	location=137192..138595
8 371Synechococcus sp JA-3-3Ab protein	BA000045.2 BAC88312.1	gene=gll0371	protein=gll0371	protein id=BAC88312.1	location=complement(385225..386628)
19 146Gloeobacter violaceus PCC7421 protein	BA000045.2 BAC88087.1	gene=glr0146	protein=glr0146	protein id=BAC88087.1	location=137192..138595
19 371Gloeobacter violaceus PCC7421 protein	BA000045.2 BAC88312.1	gene=gll0371	protein=gll0371	protein id=BAC88312.1	location=complement(385225..386628)
8 1387Synechococcus sp JA-3-3Ab protein	BA000045.2 BAC89328.1	gene=glr1387	protein=glr1387	protein id=BAC89328.1	location=1491048..1491683
8 2402Synechococcus sp JA-3-3Ab protein	BA000045.2 BAC90343.1	gene=glr2402	protein=MotA/TolQ/ExbB family proton channel protein	protein id=BAC90343.1	location=2561704..2562339
19 1387Gloeobacter violaceus PCC7421 protein	BA000045.2 BAC89328.1	gene=glr1387	protein=glr1387	protein id=BAC89328.1	location=1491048..1491683
19 2402Gloeobacter violaceus PCC7421 protein	BA000045.2 BAC90343.1	gene=glr2402	protein=MotA/TolQ/ExbB family proton channel protein	protein id=BAC90343.1	location=2561704..2562339
8 1389Synechococcus sp JA-3-3Ab protein	BA000045.2 BAC89330.1	gene=glr1389	protein=glr1389	protein id=BAC89330.1	location=1492134..1492832
8 2404Synechococcus sp JA-3-3Ab protein	BA000045.2 BAC90345.1	gene=glr2404	protein=glr2404	protein id=BAC90345.1	location=2562721..2563491
19 1389Gloeobacter violaceus PCC7421 protein	BA000045.2 BAC89330.1	gene=glr1389	protein=glr1389	protein id=BAC89330.1	location=1492134..1492832
19 2404Gloeobacter violaceus PCC7421 protein	BA000045.2 BAC90345.1	gene=glr2404	protein=glr2404	protein id=BAC90345.1	location=2562721..2563491
8 2131Synechococcus sp JA-3-3Ab protein	BA000045.2 BAC90072.1	gene=glr2131	protein=glr2131	protein id=BAC90072.1	location=2283239..2283793
8 2563Synechococcus sp JA-3-3Ab protein	BA000045.2 BAC90504.1	gene=gll2563	protein=gll2563	protein id=BAC90504.1	location=complement(2715339..2715977)
19 2131Gloeobacter violaceus PCC7421 protein	BA000045.2 BAC90072.1	gene=glr2131	protein=glr2131	protein id=BAC90072.1	location=2283239..2283793
19 2563Gloeobacter violaceus PCC7421 protein	BA000045.2 BAC90504.1	gene=gll2563	protein=gll2563	protein id=BAC90504.1	location=complement(2715339..2715977)
8 3267Synechococcus sp JA-3-3Ab protein	BA000045.2 BAC91209.1	gene=glr3268	protein=glr3268	protein id=BAC91209.1	location=3470391..3470822
8 3346Synechococcus sp JA-3-3Ab protein	BA000045.2 BAC91288.1	gene=gll3347	protein=gll3347	protein id=BAC91288.1	location=complement(3553745..3554176)
19 3267Gloeobacter violaceus PCC7421 protein	BA000045.2 BAC91209.1	gene=glr3268	protein=glr3268	protein id=BAC91209.1	location=3470391..3470822
19 3346Gloeobacter violaceus PCC7421 protein	BA000045.2 BAC91288.1	gene=gll3347	protein=gll3347	protein id=BAC91288.1	location=complement(3553745..3554176)

8 151Synechococcus sp JA-3-3Ab protein	BA000045.2 BAC88092.1	gene=gll0151	protein=gll0151	protein id=BAC88092.1	location=complement(141822..143390)
8 606Synechococcus sp JA-3-3Ab protein	BA000045.2 BAC88547.1	gene=gll0606	protein=gll0606	protein id=BAC88547.1	location=complement(649344..650912)
8 2984Synechococcus sp JA-3-3Ab protein	BA000045.2 BAC90925.1	gene=gll2984	protein=gll2984	protein id=BAC90925.1	location=complement(3183107..3184675)
8 3834Synechococcus sp JA-3-3Ab protein	BA000045.2 BAC91777.1	gene=gll3836	protein=gll3836	protein id=BAC91777.1	location=complement(4039599..4041167)
8 4320Synechococcus sp JA-3-3Ab protein	BA000045.2 BAC92263.1	gene=glr4322	protein=glr4322	protein id=BAC92263.1	location=4553924..4555492
19 151Gloeobacter violaceus PCC7421 protein	BA000045.2 BAC88092.1	gene=gll0151	protein=gll0151	protein id=BAC88092.1	location=complement(141822..143390)
19 606Gloeobacter violaceus PCC7421 protein	BA000045.2 BAC88547.1	gene=gll0606	protein=gll0606	protein id=BAC88547.1	location=complement(649344..650912)
19 2984Gloeobacter violaceus PCC7421 protein	BA000045.2 BAC90925.1	gene=gll2984	protein=gll2984	protein id=BAC90925.1	location=complement(3183107..3184675)
19 3834Gloeobacter violaceus PCC7421 protein	BA000045.2 BAC91777.1	gene=gll3836	protein=gll3836	protein id=BAC91777.1	location=complement(4039599..4041167)
19 4320Gloeobacter violaceus PCC7421 protein	BA000045.2 BAC92263.1	gene=glr4322	protein=glr4322	protein id=BAC92263.1	location=4553924..4555492
8 469Synechococcus sp JA-3-3Ab protein	BA000045.2 BAC88410.1	gene=rfbA	protein=glucose-1-phosphate thymidylyltransferase	protein id=BAC88410.1	location=502369..503244
8 1783Synechococcus sp JA-3-3Ab protein	BA000045.2 BAC89724.1	gene=gll1783	protein=glucose 1-phosphate thymidyltransferase	protein id=BAC89724.1	location=complement(1897569..1898444)
19 469Gloeobacter violaceus PCC7421 protein	BA000045.2 BAC88410.1	gene=rfbA	protein=glucose-1-phosphate thymidylyltransferase	protein id=BAC88410.1	location=502369..503244
19 1783Gloeobacter violaceus PCC7421 protein	BA000045.2 BAC89724.1	gene=gll1783	protein=glucose 1-phosphate thymidyltransferase	protein id=BAC89724.1	location=complement(1897569..1898444)
8 518Synechococcus sp JA-3-3Ab protein	BA000045.2 BAC88459.1	gene=gll0518	protein=gll0518	protein id=BAC88459.1	location=complement(556855..557763)
8 2561Synechococcus sp JA-3-3Ab protein	BA000045.2 BAC90502.1	gene=gll2561	protein=gll2561	protein id=BAC90502.1	location=complement(2714074..2714982)
8 4190Synechococcus sp JA-3-3Ab protein	BA000045.2 BAC92133.1	gene=glr4192	protein=glr4192	protein id=BAC92133.1	location=4387734..4388642
19 518Gloeobacter violaceus PCC7421 protein	BA000045.2 BAC88459.1	gene=gll0518	protein=gll0518	protein id=BAC88459.1	location=complement(556855..557763)
19 2561Gloeobacter violaceus PCC7421 protein	BA000045.2 BAC90502.1	gene=gll2561	protein=gll2561	protein id=BAC90502.1	location=complement(2714074..2714982)
19 4190Gloeobacter violaceus PCC7421 protein	BA000045.2 BAC92133.1	gene=glr4192	protein=glr4192	protein id=BAC92133.1	location=4387734..4388642
8 519Synechococcus sp JA-3-3Ab protein	BA000045.2 BAC88460.1	gene=gll0519	protein=gll0519	protein id=BAC88460.1	location=complement(557792..558193)
8 2562Synechococcus sp JA-3-3Ab protein	BA000045.2 BAC90503.1	gene=gll2562	protein=gll2562	protein id=BAC90503.1	location=complement(2715011..2715364)
8 4189Synechococcus sp JA-3-3Ab protein	BA000045.2 BAC92132.1	gene=glr4191	protein=glr4191	protein id=BAC92132.1	location=4387304..4387705
19 519Gloeobacter violaceus PCC7421 protein	BA000045.2 BAC88460.1	gene=gll0519	protein=gll0519	protein id=BAC88460.1	location=complement(557792..558193)
19 2562Gloeobacter violaceus PCC7421 protein	BA000045.2 BAC90503.1	gene=gll2562	protein=gll2562	protein id=BAC90503.1	location=complement(2715011..2715364)
19 4189Gloeobacter violaceus PCC7421 protein	BA000045.2 BAC92132.1	gene=glr4191	protein=glr4191	protein id=BAC92132.1	location=4387304..4387705
8 914Synechococcus sp JA-3-3Ab protein	BA000045.2 BAC88855.1	gene=gll0914	protein=gll0914	protein id=BAC88855.1	location=complement(969401..969739)
8 1495Synechococcus sp JA-3-3Ab protein	BA000045.2 BAC89436.1	gene=gll1495	protein=gll1495	protein id=BAC89436.1	location=complement(1608188..1608526)
19 914Gloeobacter violaceus PCC7421 protein	BA000045.2 BAC88855.1	gene=gll0914	protein=gll0914	protein id=BAC88855.1	location=complement(969401..969739)
19 1495Gloeobacter violaceus PCC7421 protein	BA000045.2 BAC89436.1	gene=gll1495	protein=gll1495	protein id=BAC89436.1	location=complement(1608188..1608526)
8 1530Synechococcus sp JA-3-3Ab protein	BA000045.2 BAC89471.1	gene=glr1530	protein=pyruvate dehydrogenase E1 component beta	protein id=BAC89471.1	location=1650902..1651885
8 2846Synechococcus sp JA-3-3Ab protein	BA000045.2 BAC90787.1	gene=glr2846	protein=pyruvate dehydrogenase E1 beta-subunit	protein id=BAC90787.1	location=3032535..3033518
19 1530Gloeobacter violaceus PCC7421 protein	BA000045.2 BAC89471.1	gene=glr1530	protein=pyruvate dehydrogenase E1 component beta	protein id=BAC89471.1	location=1650902..1651885
19 2846Gloeobacter violaceus PCC7421 protein	BA000045.2 BAC90787.1	gene=glr2846	protein=pyruvate dehydrogenase E1 beta-subunit	protein id=BAC90787.1	location=3032535..3033518
5 4345Anabaena variabilis ATCC29413 protein	CP000117.1 ABA23972.1	gene=Ava 4374	protein=conserved hypothetical protein	protein id=ABA23972.1	location=5481965..5482207
5 4346Anabaena variabilis ATCC29413 protein	CP000117.1 ABA23973.1	gene=Ava 4375	protein=conserved hypothetical protein	protein id=ABA23973.1	location=5482353..5482589
12 1930Nostoc sp PCC7120 protein	BA000019.2 BAB73632.1	gene=asl1933	protein=asl1933	protein id=BAB73632.1	location=complement(2319911..2320144)
12 1932Nostoc sp PCC7120 protein	BA000019.2 BAB73634.1	gene=asl1935	protein=asl1935	protein id=BAB73634.1	location=complement(2320597..2320830)

7 42Synechocystis sp PCC6803 protein	BA000022.2 BAA16620.1	gene=sll1397	protein=transposase		protein id=BAA16620.1	location=complement(52260..53108)
7 348Synechocystis sp PCC6803 protein	BA000022.2 BAA16927.1	gene=slr1075	protein=transposase		protein id=BAA16927.1	location=379065..379913
7 992Synechocystis sp PCC6803 protein	BA000022.2 BAA17571.1	gene=slr1357	protein=transposase		protein id=BAA17571.1	location=1098323..1099171
7 1466Synechocystis sp PCC6803 protein	BA000022.2 BAA18045.1	gene=sll1998	protein=transposase		protein id=BAA18045.1	location=complement(1623723..1624571)
7 2195Synechocystis sp PCC6803 protein	BA000022.2 BAA10164.1	gene=slr0352	protein=transposase		protein id=BAA10164.1	location=2443999..2444847
7 2265Synechocystis sp PCC6803 protein	BA000022.2 BAA10234.1	gene=slr0230	protein=transposase		protein id=BAA10234.1	location=2534106..2534954
7 2772Synechocystis sp PCC6803 protein	BA000022.2 BAA10742.1	gene=slr0704	protein=transposase		protein id=BAA10742.1	location=3097436..3098284
7 3107Synechocystis sp PCC6803 protein	BA000022.2 BAA18818.1	gene=sll0431	protein=transposase		protein id=BAA18818.1	location=complement(3512315..3513163)
14 742Microcystis aeruginosa NIES-843 protein	AP009552.1 BAG00564.1	gene=MAE 07420	protein=transposase		protein id=BAG00564.1	location=644876..645724
14 950Microcystis aeruginosa NIES-843 protein	AP009552.1 BAG00773.1	gene=MAE 09510	protein=transposase		protein id=BAG00773.1	location=complement(824160..825008)
14 1410Microcystis aeruginosa NIES-843 protein	AP009552.1 BAG01233.1	gene=MAE 14110	protein=transposase		protein id=BAG01233.1	location=1255936..1256784
14 1441Microcystis aeruginosa NIES-843 protein	AP009552.1 BAG01264.1	gene=MAE 14420	protein=transposase		protein id=BAG01264.1	location=1286000..1286848
14 2041Microcystis aeruginosa NIES-843 protein	AP009552.1 BAG01864.1	gene=MAE 20420	protein=transposase		protein id=BAG01864.1	location=complement(1829857..1830705)
14 2400Microcystis aeruginosa NIES-843 protein	AP009552.1 BAG02223.1	gene=MAE 24010	protein=transposase		protein id=BAG02223.1	location=complement(2168310..2169158)
14 2464Microcystis aeruginosa NIES-843 protein	AP009552.1 BAG02287.1	gene=MAE 24650	protein=transposase		protein id=BAG02287.1	location=2228421..2229269
14 2819Microcystis aeruginosa NIES-843 protein	AP009552.1 BAG02642.1	gene=MAE 28200	protein=transposase		protein id=BAG02642.1	location=complement(2567857..2568705)
14 3227Microcystis aeruginosa NIES-843 protein	AP009552.1 BAG03052.1	gene=MAE 32300	protein=transposase		protein id=BAG03052.1	location=2946333..2947181
14 3632Microcystis aeruginosa NIES-843 protein	AP009552.1 BAG03458.1	gene=MAE 36360	protein=transposase		protein id=BAG03458.1	location=complement(3279361..3280209)
14 3837Microcystis aeruginosa NIES-843 protein	AP009552.1 BAG03663.1	gene=MAE 38410	protein=transposase		protein id=BAG03663.1	location=complement(3471789..3472637)
14 3892Microcystis aeruginosa NIES-843 protein	AP009552.1 BAG03718.1	gene=MAE 38960	protein=transposase		protein id=BAG03718.1	location=complement(3566138..3566986)
14 4513Microcystis aeruginosa NIES-843 protein	AP009552.1 BAG04339.1	gene=MAE 45170	protein=transposase		protein id=BAG04339.1	location=4154465..4155313
14 5113Microcystis aeruginosa NIES-843 protein	AP009552.1 BAG04937.1	gene=MAE 51150	protein=transposase		protein id=BAG04937.1	location=4697263..4698111
14 5172Microcystis aeruginosa NIES-843 protein	AP009552.1 BAG04996.1	gene=MAE 51740	protein=transposase		protein id=BAG04996.1	location=complement(4746045..4746893)
14 5381Microcystis aeruginosa NIES-843 protein	AP009552.1 BAG05204.1	gene=MAE 53820	protein=transposase		protein id=BAG05204.1	location=complement(4944696..4945544)
14 5503Microcystis aeruginosa NIES-843 protein	AP009552.1 BAG05327.1	gene=MAE 55050	protein=transposase		protein id=BAG05327.1	location=complement(5080677..5081525)
14 5660Microcystis aeruginosa NIES-843 protein	AP009552.1 BAG05484.1	gene=MAE 56620	protein=transposase		protein id=BAG05484.1	location=complement(5221132..5221980)
14 5984Microcystis aeruginosa NIES-843 protein	AP009552.1 BAG05807.1	gene=MAE 59850	protein=transposase		protein id=BAG05807.1	location=5513511..5514359
1 568Cyanothece sp PCC8801 protein	CP001287.1 ACK64675.1	gene=PCC8801 0587	protein=transposase protein	IS200-family	protein id=ACK64675.1	location=615742..616155
1 813Cyanothece sp PCC8801 protein	CP001287.1 ACK64920.1	gene=PCC8801 0839	protein=transposase protein	IS200-family	protein id=ACK64920.1	location=complement(878858..879271)
1 1294Cyanothece sp PCC8801 protein	CP001287.1 ACK65401.1	gene=PCC8801 1338	protein=transposase protein	IS200-family	protein id=ACK65401.1	location=1393277..1393690
9 169Cyanothece sp PCC7424 protein	CP001291.1 ACK68643.1	gene=PCC7424 0173	protein=transposase protein	IS200-family	protein id=ACK68643.1	location=191551..191970
9 1400Cyanothece sp PCC7424 protein	CP001291.1 ACK69875.1	gene=PCC7424 1431	protein=transposase protein	IS200-family	protein id=ACK69875.1	location=complement(1574976..1575395)
9 2731Cyanothece sp PCC7424 protein	CP001291.1 ACK71206.1	gene=PCC7424 2799	protein=transposase protein	IS200-family	protein id=ACK71206.1	location=3111086..3111505

3 134Thermosynechococcus elongatus BP-1 protein	BA000039.2 BAC07687.1	gene=tlr0134	protein=tlr0134	protein id=BAC07687.1	location=114353..115579
3 205Thermosynechococcus elongatus BP-1 protein	BA000039.2 BAC07758.1	gene=tll0205	protein=tll0205	protein id=BAC07758.1	location=complement(185743..186915)
3 222Thermosynechococcus elongatus BP-1 protein	BA000039.2 BAC07775.1	gene=tll0222	protein=tll0222	protein id=BAC07775.1	location=complement(202481..203653)
3 273Thermosynechococcus elongatus BP-1 protein	BA000039.2 BAC07826.1	gene=tlr0273	protein=tlr0273	protein id=BAC07826.1	location=257476..258648
3 405Thermosynechococcus elongatus BP-1 protein	BA000039.2 BAC07958.1	gene=tlr0406	protein=tlr0406	protein id=BAC07958.1	location=403394..404566
3 511Thermosynechococcus elongatus BP-1 protein	BA000039.2 BAC08064.1	gene=tll0512	protein=tll0512	protein id=BAC08064.1	location=complement(508223..509395)
3 603Thermosynechococcus elongatus BP-1 protein	BA000039.2 BAC08156.1	gene=tll0604	protein=tll0604	protein id=BAC08156.1	location=complement(611416..612588)
3 685Thermosynechococcus elongatus BP-1 protein	BA000039.2 BAC08238.1	gene=tll0687	protein=tll0687	protein id=BAC08238.1	location=complement(706573..707799)
3 1125Thermosynechococcus elongatus BP-1 protein	BA000039.2 BAC08679.1	gene=tlr1127	protein=tlr1127	protein id=BAC08679.1	location=1159903..1161090
3 1150Thermosynechococcus elongatus BP-1 protein	BA000039.2 BAC08704.1	gene=tll1152	protein=tll1152	protein id=BAC08704.1	location=complement(1183861..1185033)
3 1372Thermosynechococcus elongatus BP-1 protein	BA000039.2 BAC08926.1	gene=tll1374	protein=tll1374	protein id=BAC08926.1	location=complement(1441028..1442200)
3 2177Thermosynechococcus elongatus BP-1 protein	BA000039.2 BAC09731.1	gene=tlr2179	protein=tlr2179	protein id=BAC09731.1	location=2263217..2264389
3 2180Thermosynechococcus elongatus BP-1 protein	BA000039.2 BAC09734.1	gene=tlr2182	protein=tlr2182	protein id=BAC09734.1	location=2266280..2267452
3 2197Thermosynechococcus elongatus BP-1 protein	BA000039.2 BAC09751.1	gene=tlr2199	protein=tlr2199	protein id=BAC09751.1	location=2283094..2284266
3 2374Thermosynechococcus elongatus BP-1 protein	BA000039.2 BAC09928.1	gene=tlr2376	protein=tlr2376	protein id=BAC09928.1	location=2486239..2487411
14 1575Microcystis aeruginosa NIES-843 protein	AP009552.1 BAG01398.1	gene=MAE 15760	protein=transposase	protein id=BAG01398.1	location=1415997..1417040
14 2940Microcystis aeruginosa NIES-843 protein	AP009552.1 BAG02765.1	gene=MAE 29430	protein=transposase	protein id=BAG02765.1	location=2681481..2682524
6 3611Arthrospira platensis NIES-39 protein	AP011615.1 BAI91453.1	gene=NIES39 J04060	protein=transposase	protein id=BAI91453.1	location=complement(3681424..3681828)
6 5325Arthrospira platensis NIES-39 protein	AP011615.1 BAI93167.1	gene=NIES39 N00500	protein=transposase	protein id=BAI93167.1	location=5473561..5473965
9 1437Cyanothece sp PCC7424 protein	CP001291.1 ACK69912.1	gene=PCC7424 1470	protein=transposase IS200-family protein	protein id=ACK69912.1	location=complement(1620393..1620800)
9 5155Cyanothece sp PCC7424 protein	CP001291.1 ACK73630.1	gene=PCC7424 5282	protein=transposase IS200-family protein	protein id=ACK73630.1	location=complement(5859213..5859620)
6 1071Arthrospira platensis NIES-39 protein	AP011615.1 BAI88913.1	gene=NIES39 C00430	protein=hypothetical protein	protein id=BAI88913.1	location=complement(1065361..1066566)
6 5156Arthrospira platensis NIES-39 protein	AP011615.1 BAI92998.1	gene=NIES39 M01610	protein=hypothetical protein	protein id=BAI92998.1	location=5245558..5246763
14 1122Microcystis aeruginosa NIES-843 protein	AP009552.1 BAG00945.1	gene=MAE 11230	protein=hypothetical protein	protein id=BAG00945.1	location=complement(974280..975422)
14 4916Microcystis aeruginosa NIES-843 protein	AP009552.1 BAG04740.1	gene=MAE 49180	protein=hypothetical protein	protein id=BAG04740.1	location=4504901..4505998
1 623Cyanothece sp PCC8801 protein	CP001287.1 ACK64730.1	gene=PCC8801 0643	protein=transposase	protein id=ACK64730.1	location=complement(670640..671638)
1 1928Cyanothece sp PCC8801 protein	CP001287.1 ACK66035.1	gene=PCC8801 1997	protein=transposase	protein id=ACK66035.1	location=2072306..2073304
14 1070Microcystis aeruginosa NIES-843 protein	AP009552.1 BAG00893.1	gene=MAE 10710	protein=transposase	protein id=BAG00893.1	location=complement(918742..919740)
14 1388Microcystis aeruginosa NIES-843 protein	AP009552.1 BAG01211.1	gene=MAE 13890	protein=transposase	protein id=BAG01211.1	location=1239551..1240408
14 1979Microcystis aeruginosa NIES-843 protein	AP009552.1 BAG01802.1	gene=MAE 19800	protein=transposase	protein id=BAG01802.1	location=1775638..1776636
14 2026Microcystis aeruginosa NIES-843 protein	AP009552.1 BAG01849.1	gene=MAE 20270	protein=transposase and inactivated derivatives	protein id=BAG01849.1	location=complement(1817962..1818960)
14 2223Microcystis aeruginosa NIES-843 protein	AP009552.1 BAG02046.1	gene=MAE 22240	protein=transposase	protein id=BAG02046.1	location=1994117..1994884
14 2341Microcystis aeruginosa NIES-843 protein	AP009552.1 BAG02164.1	gene=MAE 23420	protein=transposase	protein id=BAG02164.1	location=2108561..2109559
14 2749Microcystis aeruginosa NIES-843 protein	AP009552.1 BAG02572.1	gene=MAE 27500	protein=transposase	protein id=BAG02572.1	location=complement(2481279..2482277)
14 2882Microcystis aeruginosa NIES-843 protein	AP009552.1 BAG02707.1	gene=MAE 28850	protein=transposase	protein id=BAG02707.1	location=complement(2629909..2630907)
14 3636Microcystis aeruginosa NIES-843 protein	AP009552.1 BAG03462.1	gene=MAE 36400	protein=transposase	protein id=BAG03462.1	location=3282649..3283647
14 4403Microcystis aeruginosa NIES-843 protein	AP009552.1 BAG04229.1	gene=MAE 44070	protein=transposase	protein id=BAG04229.1	location=complement(4060904..4061902)
14 6154Microcystis aeruginosa NIES-843 protein	AP009552.1 BAG05977.1	gene=MAE 61550	protein=transposase	protein id=BAG05977.1	location=5699143..5700141

9 1181Cyanothece sp PCC7424 protein	CP001291.1 ACK69656.1	gene=PCC7424 1208	protein=Insertion element protein	protein id=ACK69656.1	location=join(1322136..1322465,1322467..1322829)
9 2458Cyanothece sp PCC7424 protein	CP001291.1 ACK70933.1	gene=PCC7424 2516	protein=Insertion element protein	protein id=ACK70933.1	location=join(2799135..2799464,2799466..2799828)
9 5076Cyanothece sp PCC7424 protein	CP001291.1 ACK73551.1	gene=PCC7424 5203	protein=Insertion element protein	protein id=ACK73551.1	location=complement(join(5775088..5775450,5775452..5775781))
16 695Acharyochloris marina MBIC11017 protein	CP000828.1 ABW25786.1	gene=AM1 0742	protein=IS1 transposase	protein id=ABW25786.1	location=720392..721081
16 3378Acharyochloris marina MBIC11017 protein	CP000828.1 ABW28467.1	gene=AM1 3475	protein=IS1 transposase	protein id=ABW28467.1	location=complement(3510465..3511154)
16 5827Acharyochloris marina MBIC11017 protein	CP000828.1 ABW30914.1	gene=AM1 5978	protein=IS1 transposase	protein id=ABW30914.1	location=6055468..6056157
1 1064Cyanothece sp PCC8801 protein	CP001287.1 ACK65171.1	gene=PCC8801 1098	protein=transposase, IS605 OrfB family	protein id=ACK65171.1	location=complement(1157486..1158697)
1 4021Cyanothece sp PCC8801 protein	CP001287.1 ACK68129.1	gene=PCC8801 4201	protein=transposase, IS605 OrfB family	protein id=ACK68129.1	location=4404944..4406155
1 4249Cyanothece sp PCC8801 protein	CP001287.1 ACK68357.1	gene=PCC8801 4435	protein=transposase, IS605 OrfB family	protein id=ACK68357.1	location=complement(4663329..4664540)
3 257Thermosynechococcus elongatus BP-1 protein	BA000039.2 BAC07810.1	gene=tlr0257	protein=tlr0257	protein id=BAC07810.1	location=241585..242763
3 400Thermosynechococcus elongatus BP-1 protein	BA000039.2 BAC07953.1	gene=tlr0401	protein=tlr0401	protein id=BAC07953.1	location=397671..398849
3 1378Thermosynechococcus elongatus BP-1 protein	BA000039.2 BAC08932.1	gene=tlr1380	protein=tlr1380	protein id=BAC08932.1	location=1447751..1448929
3 2236Thermosynechococcus elongatus BP-1 protein	BA000039.2 BAC09790.1	gene=tlr2238	protein=tlr2238	protein id=BAC09790.1	location=2320881..2322059
16 218Acharyochloris marina MBIC11017 protein	CP000828.1 ABW25309.1	gene=AM1 0223	protein=transposase	protein id=ABW25309.1	location=complement(219734..221044)
16 496Acharyochloris marina MBIC11017 protein	CP000828.1 ABW25587.1	gene=AM1 0535	protein=transposase	protein id=ABW25587.1	location=515940..517250
16 637Acharyochloris marina MBIC11017 protein	CP000828.1 ABW25728.1	gene=AM1 0681	protein=transposase	protein id=ABW25728.1	location=666645..667955
16 916Acharyochloris marina MBIC11017 protein	CP000828.1 ABW26007.1	gene=AM1 0965	protein=transposase	protein id=ABW26007.1	location=940229..941539
16 1073Acharyochloris marina MBIC11017 protein	CP000828.1 ABW26164.1	gene=AM1 1125	protein=transposase	protein id=ABW26164.1	location=1100397..1101707
16 1854Acharyochloris marina MBIC11017 protein	CP000828.1 ABW26945.1	gene=AM1 1927	protein=transposase	protein id=ABW26945.1	location=complement(1922983..1924293)
16 2315Acharyochloris marina MBIC11017 protein	CP000828.1 ABW27406.1	gene=AM1 2396	protein=transposase	protein id=ABW27406.1	location=complement(2402873..2404183)
16 2506Acharyochloris marina MBIC11017 protein	CP000828.1 ABW27597.1	gene=AM1 2590	protein=transposase	protein id=ABW27597.1	location=2629004..2630314
16 2509Acharyochloris marina MBIC11017 protein	CP000828.1 ABW27600.1	gene=AM1 2593	protein=transposase	protein id=ABW27600.1	location=complement(2631483..2632793)
16 3530Acharyochloris marina MBIC11017 protein	CP000828.1 ABW28619.1	gene=AM1 3629	protein=transposase	protein id=ABW28619.1	location=complement(3687534..3688844)
16 3602Acharyochloris marina MBIC11017 protein	CP000828.1 ABW28691.1	gene=AM1 3701	protein=transposase	protein id=ABW28691.1	location=complement(3758553..3759863)
16 3821Acharyochloris marina MBIC11017 protein	CP000828.1 ABW28909.1	gene=AM1 3924	protein=transposase	protein id=ABW28909.1	location=3975226..3976536
16 4123Acharyochloris marina MBIC11017 protein	CP000828.1 ABW29211.1	gene=AM1 4231	protein=transposase	protein id=ABW29211.1	location=4250628..4251938
16 5052Acharyochloris marina MBIC11017 protein	CP000828.1 ABW30139.1	gene=AM1 5177	protein=transposase	protein id=ABW30139.1	location=complement(5241022..5242332)
16 5160Acharyochloris marina MBIC11017 protein	CP000828.1 ABW30247.1	gene=AM1 5286	protein=transposase	protein id=ABW30247.1	location=5352546..5353856
16 5539Acharyochloris marina MBIC11017 protein	CP000828.1 ABW30626.1	gene=AM1 5679	protein=transposase	protein id=ABW30626.1	location=complement(5743964..5745274)
16 6106Acharyochloris marina MBIC11017 protein	CP000828.1 ABW31193.1	gene=AM1 6261	protein=transposase	protein id=ABW31193.1	location=complement(6336950..6338260)
17 2423Nostoc punctiforme PCC73102 protein	CP001037.1 ACC81227.1	gene=Npun F2690	protein=Transposase-like protein	protein id=ACC81227.1	location=3341372..3342682
17 2839Nostoc punctiforme PCC73102 protein	CP001037.1 ACC81643.1	gene=Npun F3186	protein=Transposase-like protein	protein id=ACC81643.1	location=3966579..3967889
17 2974Nostoc punctiforme PCC73102 protein	CP001037.1 ACC81778.1	gene=Npun F3348	protein=Transposase-like protein	protein id=ACC81778.1	location=4168700..4170010
17 4991Nostoc punctiforme PCC73102 protein	CP001037.1 ACC83796.1	gene=Npun F5490	protein=Transposase-like protein	protein id=ACC83796.1	location=6779978..6781288
1 1753Cyanothece sp PCC8801 protein	CP001287.1 ACK65860.1	gene=PCC8801 1816	protein=transposase IS204/IS1001/IS1096/IS1165 family protein	protein id=ACK65860.1	location=complement(1898016..1899242)
1 3366Cyanothece sp PCC8801 protein	CP001287.1 ACK67474.1	gene=PCC8801 3509	protein=transposase IS204/IS1001/IS1096/IS1165 family protein	protein id=ACK67474.1	location=3661150..3662376
1 3865Cyanothece sp PCC8801 protein	CP001287.1 ACK67973.1	gene=PCC8801 4034	protein=transposase IS204/IS1001/IS1096/IS1165 family protein	protein id=ACK67973.1	location=complement(4225337..4226563)
14 2469Microcystis aeruginosa NIES-843 protein	AP009552.1 BAG02292.1	gene=MAE 24700	protein=transposase	protein id=BAG02292.1	location=complement(2233792..2234811)
14 2696Microcystis aeruginosa NIES-843 protein	AP009552.1 BAG02519.1	gene=MAE 26970	protein=transposase	protein id=BAG02519.1	location=complement(2436880..2438094)

6 1824Arthrospira platensis NIES-39 protein	AP011615.1 BAI89666.1	gene=NIES39 D02460	protein=putative transposase	protein id=BAI89666.1	location=complement(1848140..1848499)
6 4845Arthrospira platensis NIES-39 protein	AP011615.1 BAI92687.1	gene=NIES39 L05300	protein=putative transposase	protein id=BAI92687.1	location=4909208..4909567
16 451Acharyochloris marina MBIC11017 protein	CP000828.1 ABW25542.1	gene=AM1 0489	protein=transposase, putative	protein id=ABW25542.1	location=469018..469401
16 463Acharyochloris marina MBIC11017 protein	CP000828.1 ABW25554.1	gene=AM1 0501	protein=transposase, putative	protein id=ABW25554.1	location=complement(477589..477972)
16 2017Acharyochloris marina MBIC11017 protein	CP000828.1 ABW27108.1	gene=AM1 2093	protein=transposase, putative	protein id=ABW27108.1	location=2085736..2086095
14 512Microcystis aeruginosa NIES-843 protein	AP009552.1 BAG00334.1	gene=MAE 05120	protein=transposase	protein id=BAG00334.1	location=456375..457418
14 748Microcystis aeruginosa NIES-843 protein	AP009552.1 BAG00570.1	gene=MAE 07480	protein=transposase	protein id=BAG00570.1	location=649201..650244
14 1030Microcystis aeruginosa NIES-843 protein	AP009552.1 BAG00853.1	gene=MAE 10310	protein=transposase	protein id=BAG00853.1	location=complement(884964..886007)
14 4798Microcystis aeruginosa NIES-843 protein	AP009552.1 BAG04622.1	gene=MAE 48000	protein=transposase	protein id=BAG04622.1	location=4401177..4402220
16 124Acharyochloris marina MBIC11017 protein	CP000828.1 ABW25215.1	gene=AM1 0129	protein=hypothetical protein	protein id=ABW25215.1	location=complement(128563..129672)
16 155Acharyochloris marina MBIC11017 protein	CP000828.1 ABW25246.1	gene=AM1 0160	protein=hypothetical protein	protein id=ABW25246.1	location=158975..160084
16 1273Acharyochloris marina MBIC11017 protein	CP000828.1 ABW26364.1	gene=AM1 1330	protein=hypothetical protein	protein id=ABW26364.1	location=1313003..1314112
16 4169Acharyochloris marina MBIC11017 protein	CP000828.1 ABW29257.1	gene=AM1 4277	protein=hypothetical protein	protein id=ABW29257.1	location=complement(4296768..4297877)
16 4971Acharyochloris marina MBIC11017 protein	CP000828.1 ABW30058.1	gene=AM1 5094	protein=hypothetical protein	protein id=ABW30058.1	location=5147289..5148398
16 5777Acharyochloris marina MBIC11017 protein	CP000828.1 ABW30864.1	gene=AM1 5926	protein=hypothetical protein	protein id=ABW30864.1	location=5992224..5993333
16 6105Acharyochloris marina MBIC11017 protein	CP000828.1 ABW31192.1	gene=AM1 6260	protein=hypothetical protein	protein id=ABW31192.1	location=6335818..6336927
14 165Microcystis aeruginosa NIES-843 protein	AP009552.1 BAF99986.1	gene=MAE 01650	protein=hypothetical protein	protein id=BAF99986.1	location=133753..134526
14 3306Microcystis aeruginosa NIES-843 protein	AP009552.1 BAG03133.1	gene=MAE 33110	protein=hypothetical protein	protein id=BAG03133.1	location=complement(3016288..3017061)
14 3422Microcystis aeruginosa NIES-843 protein	AP009552.1 BAG03248.1	gene=MAE 34260	protein=hypothetical protein	protein id=BAG03248.1	location=complement(3114659..3115432)
14 4305Microcystis aeruginosa NIES-843 protein	AP009552.1 BAG04131.1	gene=MAE 43090	protein=hypothetical protein	protein id=BAG04131.1	location=3971211..3971984
17 1920Nostoc punctiforme PCC73102 protein	CP001037.1 ACC80724.1	gene=Npun F2107	protein=conserved hypothetical protein	protein id=ACC80724.1	location=2576706..2577554
17 4319Nostoc punctiforme PCC73102 protein	CP001037.1 ACC83123.1	gene=Npun R4774	protein=conserved hypothetical protein	protein id=ACC83123.1	location=complement(5915257..5916105)
9 3766Cyanothece sp PCC7424 protein	CP001291.1 ACK72241.1	gene=PCC7424 3862	protein=hypothetical protein	protein id=ACK72241.1	location=complement(join(4297108..4297866,4297868..4297930))
9 3802Cyanothece sp PCC7424 protein	CP001291.1 ACK72277.1	gene=PCC7424 3899	protein=transposase IS702 family protein	protein id=ACK72277.1	location=4332844..4333587

14	484	Microcystis aeruginosa NIES-843 protein	AP009552.1 BAG00306.1	gene=MAE 04840	protein=transposase	protein id=BAG00306.1	location=431565..432356
14	783	Microcystis aeruginosa NIES-843 protein	AP009552.1 BAG00605.1	gene=MAE 07830	protein=transposase	protein id=BAG00605.1	location=679549..680406
14	895	Microcystis aeruginosa NIES-843 protein	AP009552.1 BAG00718.1	gene=MAE 08960	protein=transposase	protein id=BAG00718.1	location=778709..779566
14	928	Microcystis aeruginosa NIES-843 protein	AP009552.1 BAG00751.1	gene=MAE 09290	protein=transposase	protein id=BAG00751.1	location=803601..804458
14	990	Microcystis aeruginosa NIES-843 protein	AP009552.1 BAG00813.1	gene=MAE 09910	protein=transposase	protein id=BAG00813.1	location=complement(859757..860614)
14	1086	Microcystis aeruginosa NIES-843 protein	AP009552.1 BAG00909.1	gene=MAE 10870	protein=transposase	protein id=BAG00909.1	location=complement(933528..934385)
14	2071	Microcystis aeruginosa NIES-843 protein	AP009552.1 BAG01894.1	gene=MAE 20720	protein=transposase	protein id=BAG01894.1	location=1848286..1849143
14	2319	Microcystis aeruginosa NIES-843 protein	AP009552.1 BAG02142.1	gene=MAE 23200	protein=transposase	protein id=BAG02142.1	location=2084969..2085826
14	2606	Microcystis aeruginosa NIES-843 protein	AP009552.1 BAG02429.1	gene=MAE 26070	protein=transposase	protein id=BAG02429.1	location=complement(2356803..2357660)
14	2710	Microcystis aeruginosa NIES-843 protein	AP009552.1 BAG02533.1	gene=MAE 27110	protein=transposase	protein id=BAG02533.1	location=complement(2448484..2449323)
14	3325	Microcystis aeruginosa NIES-843 protein	AP009552.1 BAG03152.1	gene=MAE 33300	protein=transposase	protein id=BAG03152.1	location=complement(3028164..3029021)
14	3360	Microcystis aeruginosa NIES-843 protein	AP009552.1 BAG03187.1	gene=MAE 33650	protein=transposase	protein id=BAG03187.1	location=3065824..3066591
14	3439	Microcystis aeruginosa NIES-843 protein	AP009552.1 BAG03265.1	gene=MAE 34430	protein=transposase	protein id=BAG03265.1	location=complement(3127634..3128401)
14	3839	Microcystis aeruginosa NIES-843 protein	AP009552.1 BAG03665.1	gene=MAE 38430	protein=transposase	protein id=BAG03665.1	location=3473455..3474312
14	3927	Microcystis aeruginosa NIES-843 protein	AP009552.1 BAG03753.1	gene=MAE 39310	protein=transposase	protein id=BAG03753.1	location=3616333..3617190
14	3986	Microcystis aeruginosa NIES-843 protein	AP009552.1 BAG03812.1	gene=MAE 39900	protein=transposase	protein id=BAG03812.1	location=3663197..3664054
14	4266	Microcystis aeruginosa NIES-843 protein	AP009552.1 BAG04092.1	gene=MAE 42700	protein=transposase	protein id=BAG04092.1	location=complement(3938799..3939656)
14	4843	Microcystis aeruginosa NIES-843 protein	AP009552.1 BAG04667.1	gene=MAE 48450	protein=transposase	protein id=BAG04667.1	location=4447164..4448021
14	5305	Microcystis aeruginosa NIES-843 protein	AP009552.1 BAG05128.1	gene=MAE 53060	protein=transposase	protein id=BAG05128.1	location=complement(4868782..4869639)
14	5415	Microcystis aeruginosa NIES-843 protein	AP009552.1 BAG05239.1	gene=MAE 54170	protein=transposase	protein id=BAG05239.1	location=complement(4978973..4979830)
14	5699	Microcystis aeruginosa NIES-843 protein	AP009552.1 BAG05523.1	gene=MAE 57010	protein=transposase	protein id=BAG05523.1	location=5261959..5262816
14	6145	Microcystis aeruginosa NIES-843 protein	AP009552.1 BAG05968.1	gene=MAE 61460	protein=transposase	protein id=BAG05968.1	location=complement(5692792..5693649)
14	6233	Microcystis aeruginosa NIES-843 protein	AP009552.1 BAG06056.1	gene=MAE 62340	protein=transposase	protein id=BAG06056.1	location=complement(5773659..5774516)
14	6284	Microcystis aeruginosa NIES-843 protein	AP009552.1 BAG06108.1	gene=MAE 62860	protein=transposase	protein id=BAG06108.1	location=5820820..5821677
14	1381	Microcystis aeruginosa NIES-843 protein	AP009552.1 BAG01204.1	gene=MAE 13820	protein=transposase	protein id=BAG01204.1	location=1236257..1236883
14	2091	Microcystis aeruginosa NIES-843 protein	AP009552.1 BAG01914.1	gene=MAE 20920	protein=transposase	protein id=BAG01914.1	location=complement(1867658..1868284)
16	1185	Acharyochloris marina MBIC11017 protein	CP000828.1 ABW26276.1	gene=AM1 1238	protein=transposase, putative	protein id=ABW26276.1	location=complement(1225141..1225770)
16	4321	Acharyochloris marina MBIC11017 protein	CP000828.1 ABW29409.1	gene=AM1 4432	protein=transposase, putative	protein id=ABW29409.1	location=complement(4460718..4461347)
14	145	Microcystis aeruginosa NIES-843 protein	AP009552.1 BAF99966.1	gene=MAE 01450	protein=transposase	protein id=BAF99966.1	location=120122..120571
14	2092	Microcystis aeruginosa NIES-843 protein	AP009552.1 BAG01915.1	gene=MAE 20930	protein=transposase	protein id=BAG01915.1	location=complement(1868347..1868796)
16	716	Acharyochloris marina MBIC11017 protein	CP000828.1 ABW25807.1	gene=AM1 0763	protein=transposase, putative	protein id=ABW25807.1	location=complement(741568..742089)
16	1481	Acharyochloris marina MBIC11017 protein	CP000828.1 ABW26572.1	gene=AM1 1547	protein=transposase, putative	protein id=ABW26572.1	location=1528937..1529458
16	1545	Acharyochloris marina MBIC11017 protein	CP000828.1 ABW26636.1	gene=AM1 1612	protein=transposase, putative	protein id=ABW26636.1	location=1584889..1585410

12 3982Nostoc sp PCC7120 protein	BA000019.2 BAB75685.1	gene=all3986	protein=transposase	protein id=BAB75685.1	location=complement(4801437..4802645)
12 4100Nostoc sp PCC7120 protein	BA000019.2 BAB75803.1	gene=alr4104	protein=transposase	protein id=BAB75803.1	location=4944752..4945960
14 530Microcystis aeruginosa NIES-843 protein	AP009552.1 BAG00352.1	gene=MAE 05300	protein=transposase	protein id=BAG00352.1	location=471056..472243
14 4484Microcystis aeruginosa NIES-843 protein	AP009552.1 BAG04310.1	gene=MAE 44880	protein=transposase	protein id=BAG04310.1	location=complement(4126673..4127860)
1 364Cyanothece sp PCC8801 protein	CP001287.1 ACK64471.1	gene=PCC8801 0373	protein=putative transposase IS891/IS1136/IS1341 family	protein id=ACK64471.1	location=371253..372587
1 3436Cyanothece sp PCC8801 protein	CP001287.1 ACK67544.1	gene=PCC8801 3581	protein=putative transposase IS891/IS1136/IS1341 family	protein id=ACK67544.1	location=complement(3730986..3732320)
9 804Cyanothece sp PCC7424 protein	CP001291.1 ACK69278.1	gene=PCC7424 0822	protein=putative transposase IS605 family	protein id=ACK69278.1	location=complement(910819..912054)
9 3156Cyanothece sp PCC7424 protein	CP001291.1 ACK71631.1	gene=PCC7424 3231	protein=putative transposase IS605 family	protein id=ACK71631.1	location=3615830..3617065
