# Supplementary material for: Gene copy number variation and its significance in cyanobacterial phylogeny
Source: BMC Microbiol. 2012 Aug 15;12:177. doi: 10.1186/1471-2180-12-177 (PMC3552681; doi:10.1186/1471-2180-12-177)
Supplement: Additional file 2 — 16S rRNA gene copy data including data from the rrndb-database. Table with information on 16S rRNA copy numbers including data received from the rrnDB database [45] marked (*). [file 1471-2180-12-177-S2.pdf]

species	Group	# of 16S rRNA copies
Acharyochloris marina MBIC11017	G1	2
Anabaena variabilis ATCC 29413	G3	4
Arthrospira platensis NIES 39	G1	2
Cyanothece sp. PCC 7424	G1	3
Cyanothece sp. PCC 8801	G1	2
Gloeobacter violaceus PCC 7421	G0	1
Microcystis aeruginosa NIES-843	G1	2
Nostoc azollae 0708	G3	4
Nostoc punctiforme PCC 73102	G3	4
Nostoc sp. PCC 7120	G3	4
Prochlorococcus marinus str. MIT 9211	G0	1
Prochlorococcus marinus str. MIT 9303	G0	2
P. marinus subsp. pastoris str. CCMP1986	G0	1
Synechococcus elongatus PCC 6301	G1	2
Synechococcus sp. PCC 7002	G1	2
Synechococcus sp. RCC307	G1	1
Synechococcus sp. WH 7803	G1	2
Thermosynechococcus elongatus BP-1	G1	1
Trichodesmium erythraeum IMS101	G2	2
cyanobacterium UCYN-A	G0	2
Oscillatoria sp PCC 65061*	G1	1
Aphanizomenon os-aquae PCC 79051*	G3	5
Anabaenopsis PCC 92151*	G3	4
Anabaena os-aquae PCC 93021*	G3	5
Nodularia PCC 93501*	G3	4
Anabaenopsis elenkinii PCC 94201*	G3	4
Cyanospira rippkae PCC 95011*	G3	4
Cyanospira capsulata PCC 95021*	G3	4
