# Supplementary figures and images for: Gene copy number variation and its significance in cyanobacterial phylogeny
Source: BMC Microbiol. 2012 Aug 15;12:177. doi: 10.1186/1471-2180-12-177 (PMC3552681; doi:10.1186/1471-2180-12-177)

16S rRNA gene (incl. additional data from rrndb)  $\rho=0.87$ ,  $R=0.902$

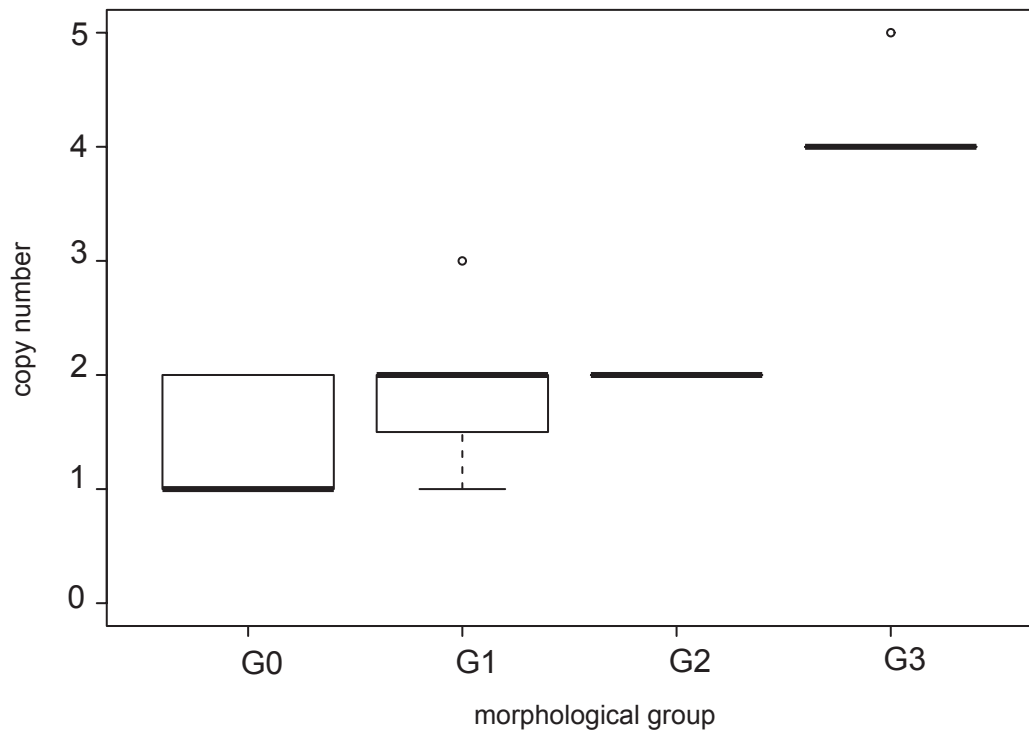

Supplement: Additional file 3 — Distribution of 16S rRNA copy numbers using additional data from rrndb3. Boxplot representations of the 16S rRNA gene copy number distribution across the previously defined morphological groups. Additional data on 16S rRNA copy numbers were received from the rrndb-database [45]. Spearman’s rank correlation coefficient (ρ) and Pearson’s correlation coefficient (R) are displayed above the graph. A strong correlation of 16S rRNA gene copies to terminally differentiated cyanobacteria is supported. [file 1471-2180-12-177-S3.pdf]

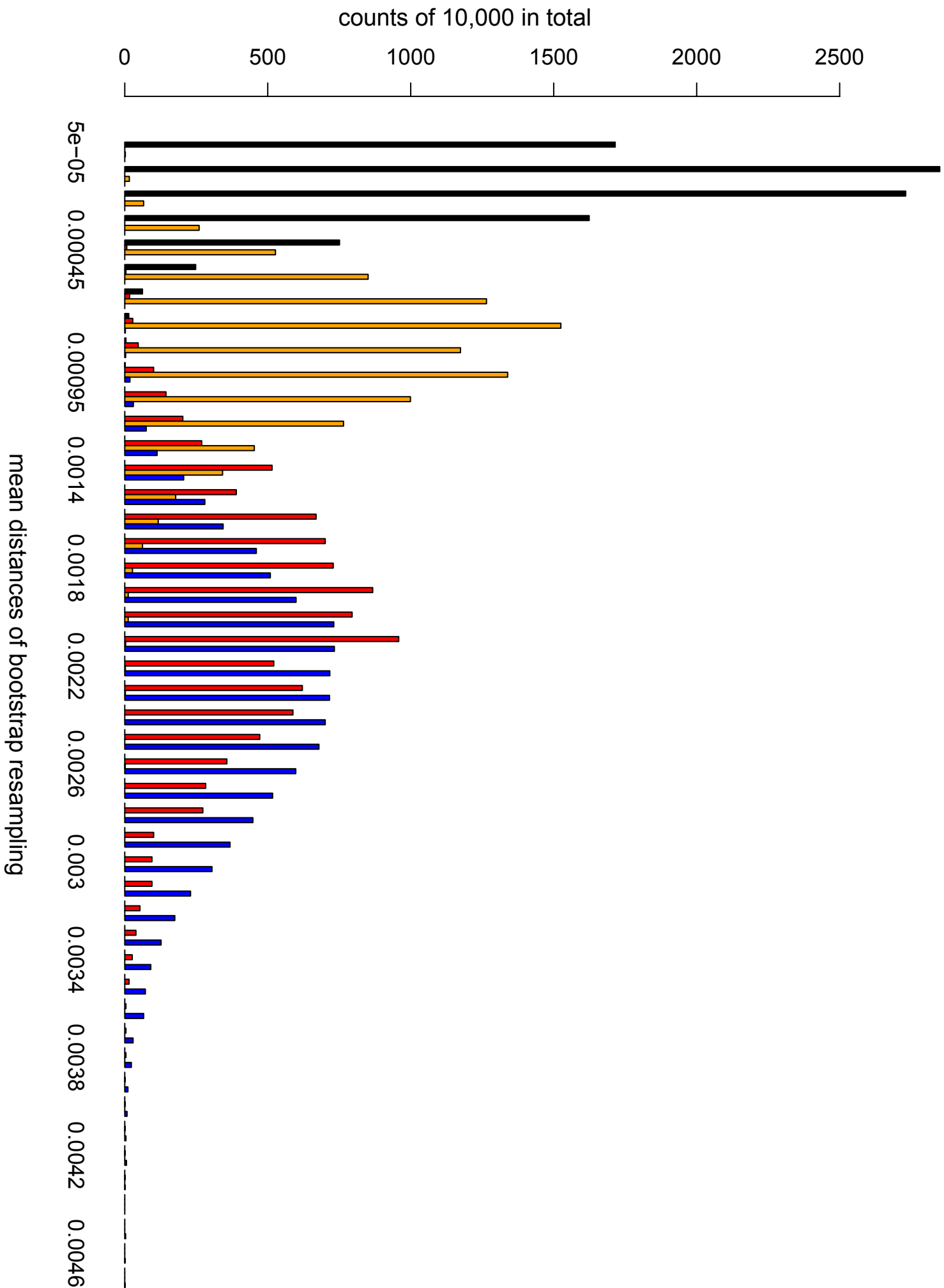

Supplement: Additional file 4 — Distribution of mean distances within species of bootstrap samples for the different eubacterial phyla. The distribution of mean distances of the bootstrap samples presented as a histogram. The 95% confidence intervals between cyanobacteria and Chloroflexi, Spirochaetes and Bacteroidetes do not overlap. Cyanobacterial 16S rRNA gene sequence variation within species is significantly lower. [file 1471-2180-12-177-S4.pdf]

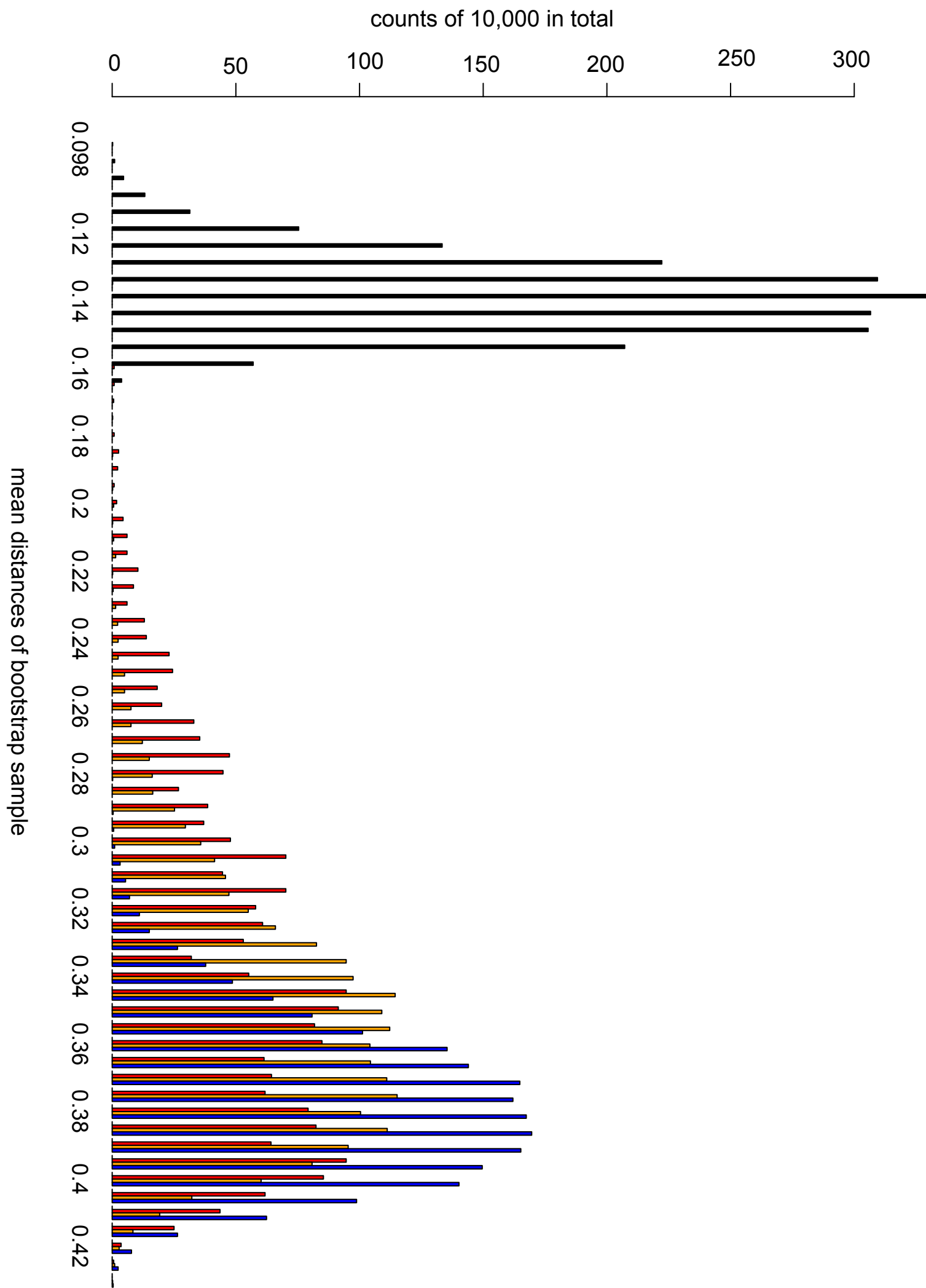

Supplement: Additional file 5 — Distribution of mean distances between species of bootstrap samples for the different eubacterial phyla. The distribution of mean distances of the bootstrap samples presented as a histogram. The 95% confidence intervals between cyanobacteria and the other eubacterial phyla do not overlap. Cyanobacterial 16S rRNA gene sequence variation between species are significantly lower. [file 1471-2180-12-177-S5.pdf]

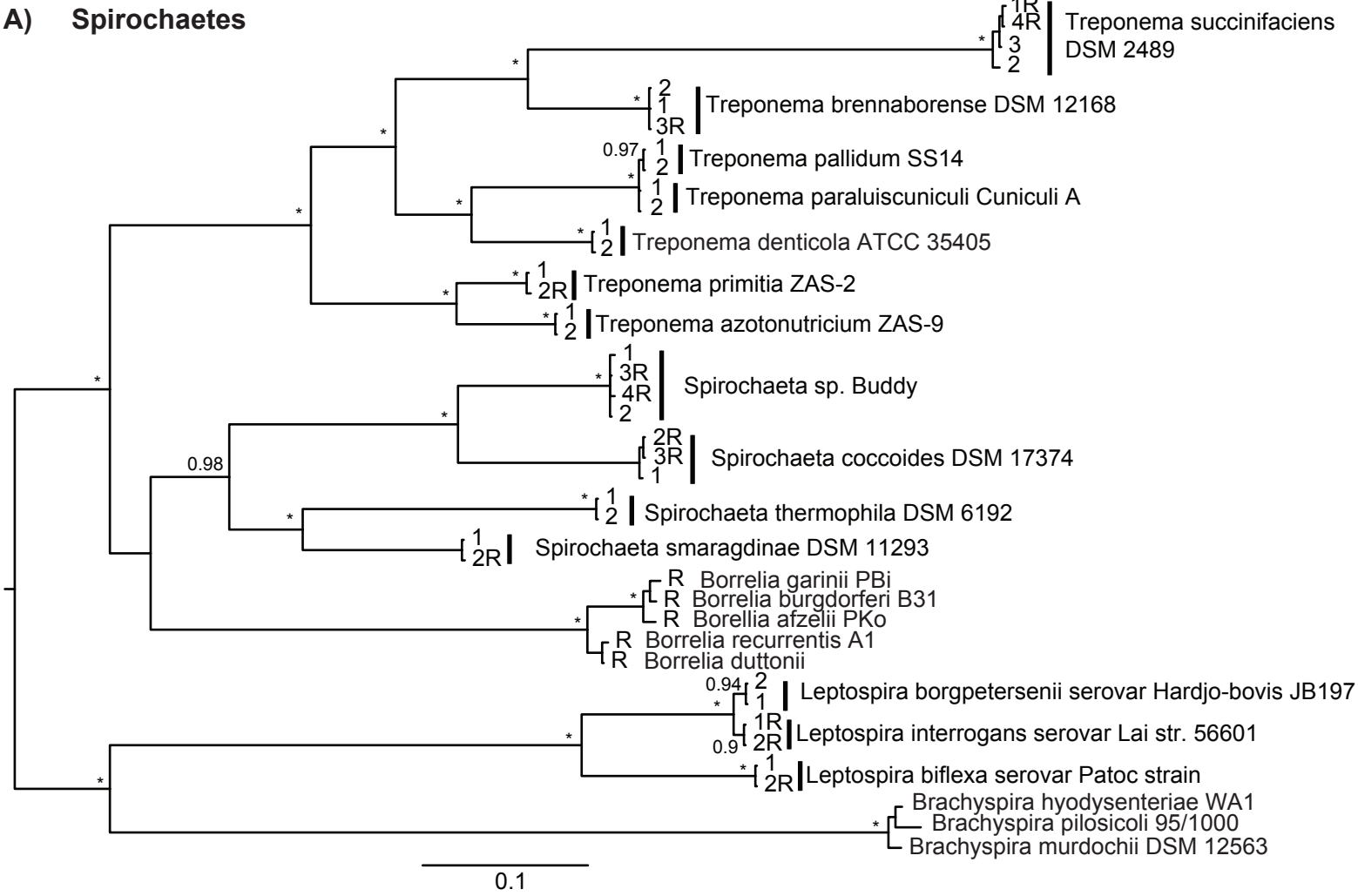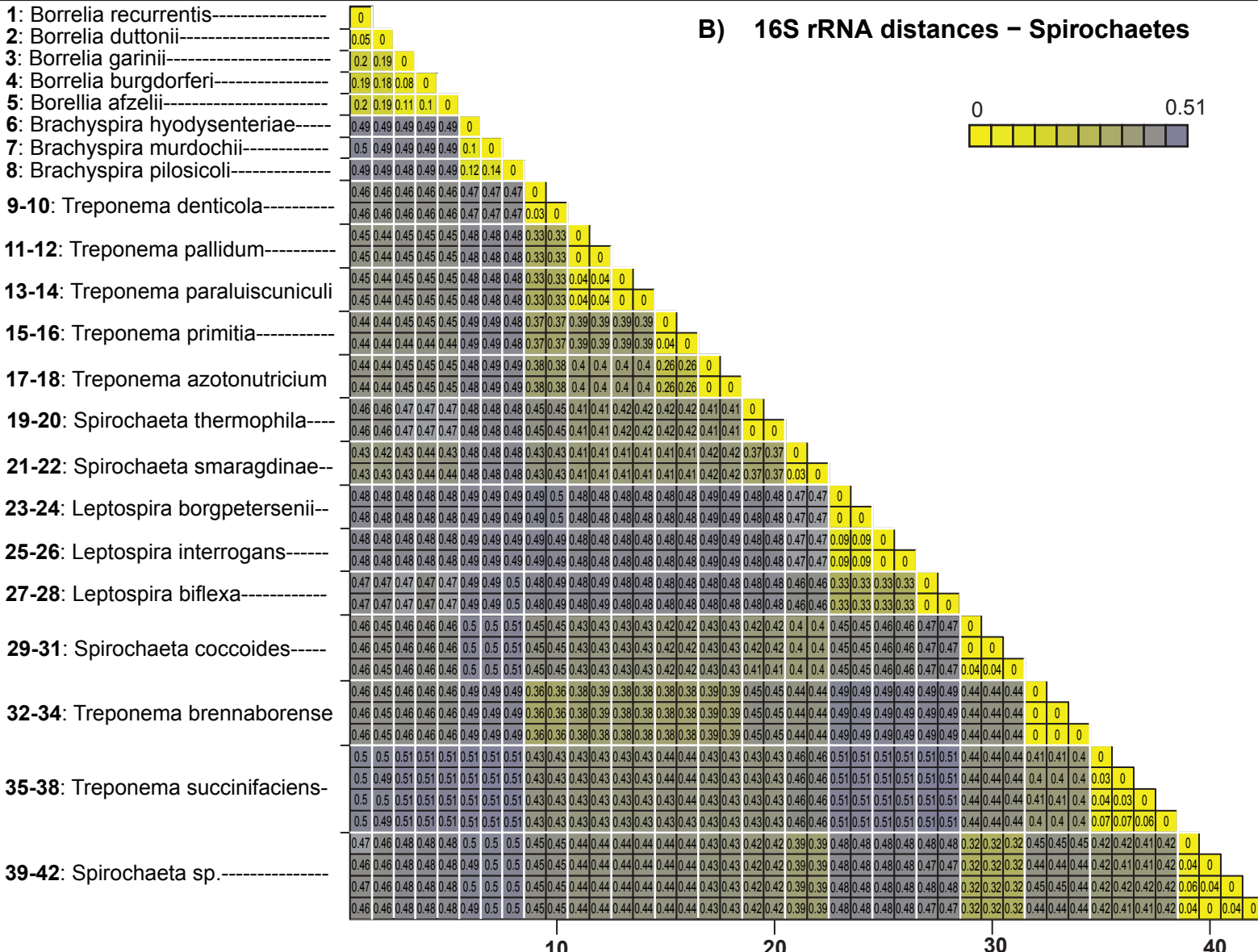

Supplement: Additional file 6 — Phylogenetic tree and distance matrix of Spirochaetes. (A) Phylogenetic tree of the eubacterial phylum Spirochaetes including all 16S rRNA gene copies, reconstructed using Bayesian analysis. On the nodes posterior probabilities >0.90 are displayed. The letter “R” denote gene copies that are positioned on the reverse DNA strand. (B) Distance matrix of Spirochaetes. Genetic distances have been estimated according to the K80 substitution model. White lines separate sequence copies of different species. [file 1471-2180-12-177-S6.pdf]

Bacteroidetes

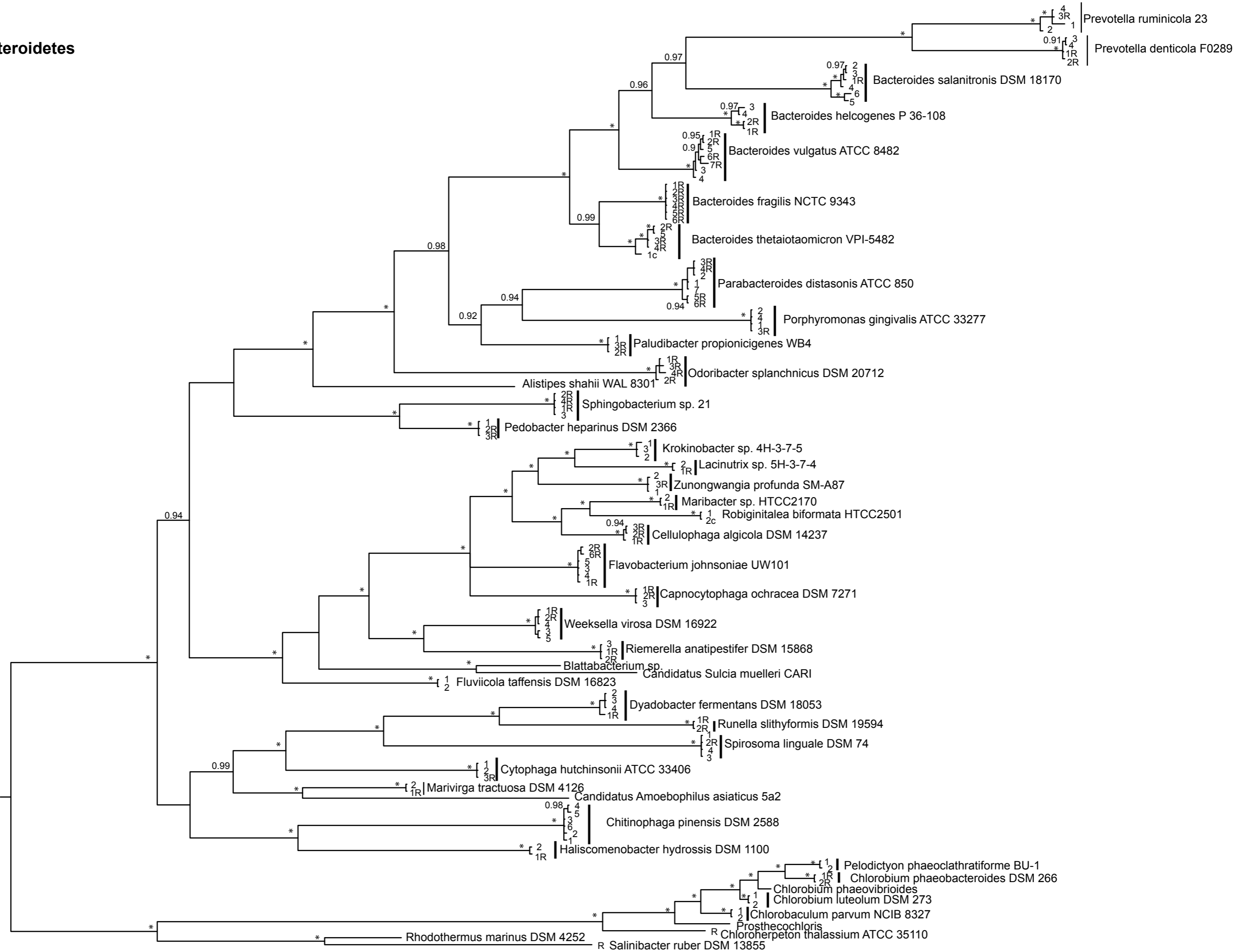

0.2

Supplement: Additional file 7 — Phylogenetic tree of Bacteroidetes. Phylogenetic tree of the eubacterial phylum Bacteroidetes including all 16S rRNA gene copies, reconstructed using Bayesian analysis. On the nodes posterior probabilities >0.90 are displayed.The letter “R” denote gene copies that are positioned on the reverse DNA strand. [file 1471-2180-12-177-S7.pdf]

Distance matrix of Bacteroidetes

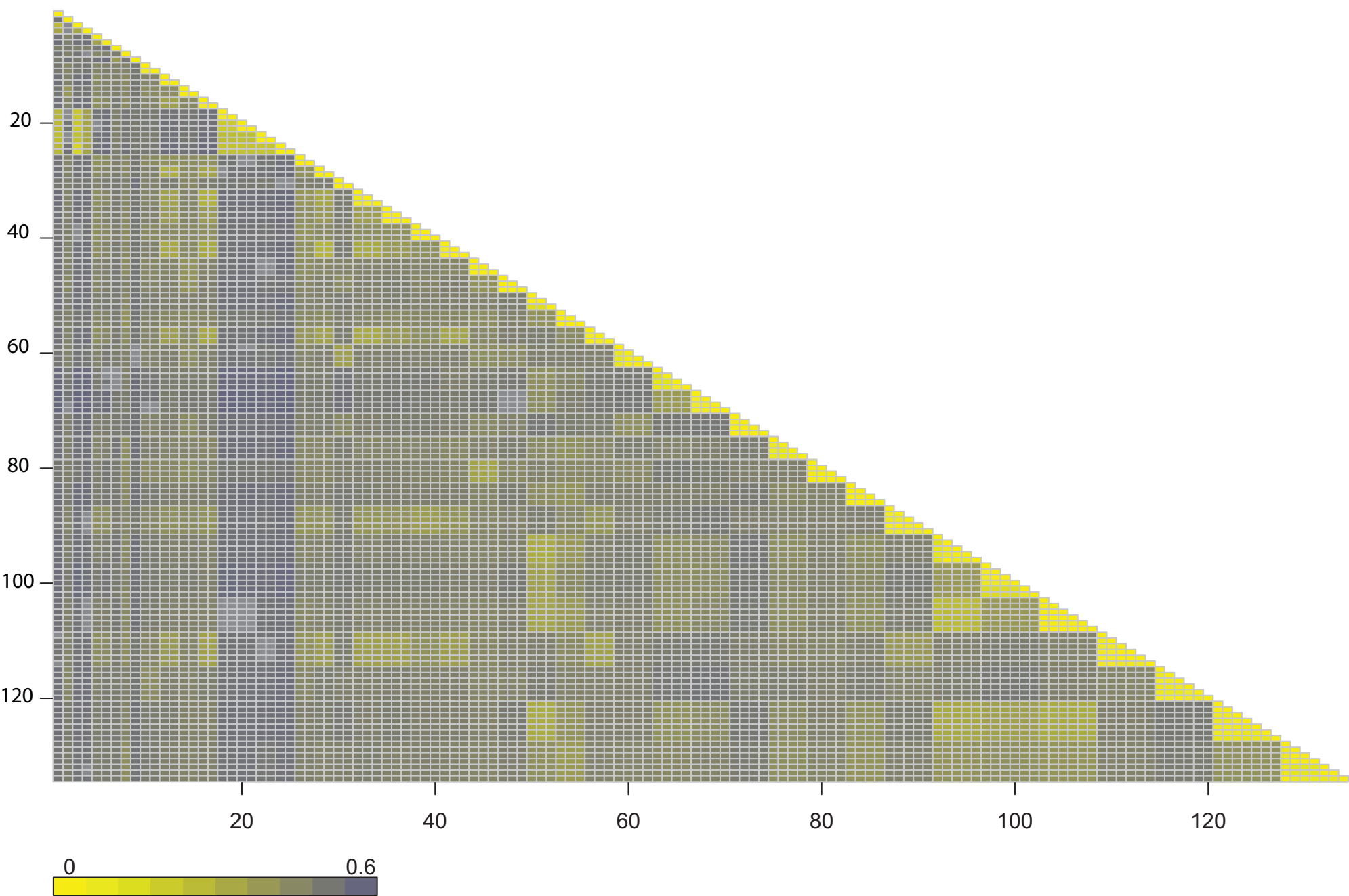

Supplement: Additional file 8 — Distance matrix of Bacteroidetes. Genetic distances have been estimated according to the K80 substitution model. White lines separate sequence copies of different species. [file 1471-2180-12-177-S8.pdf]

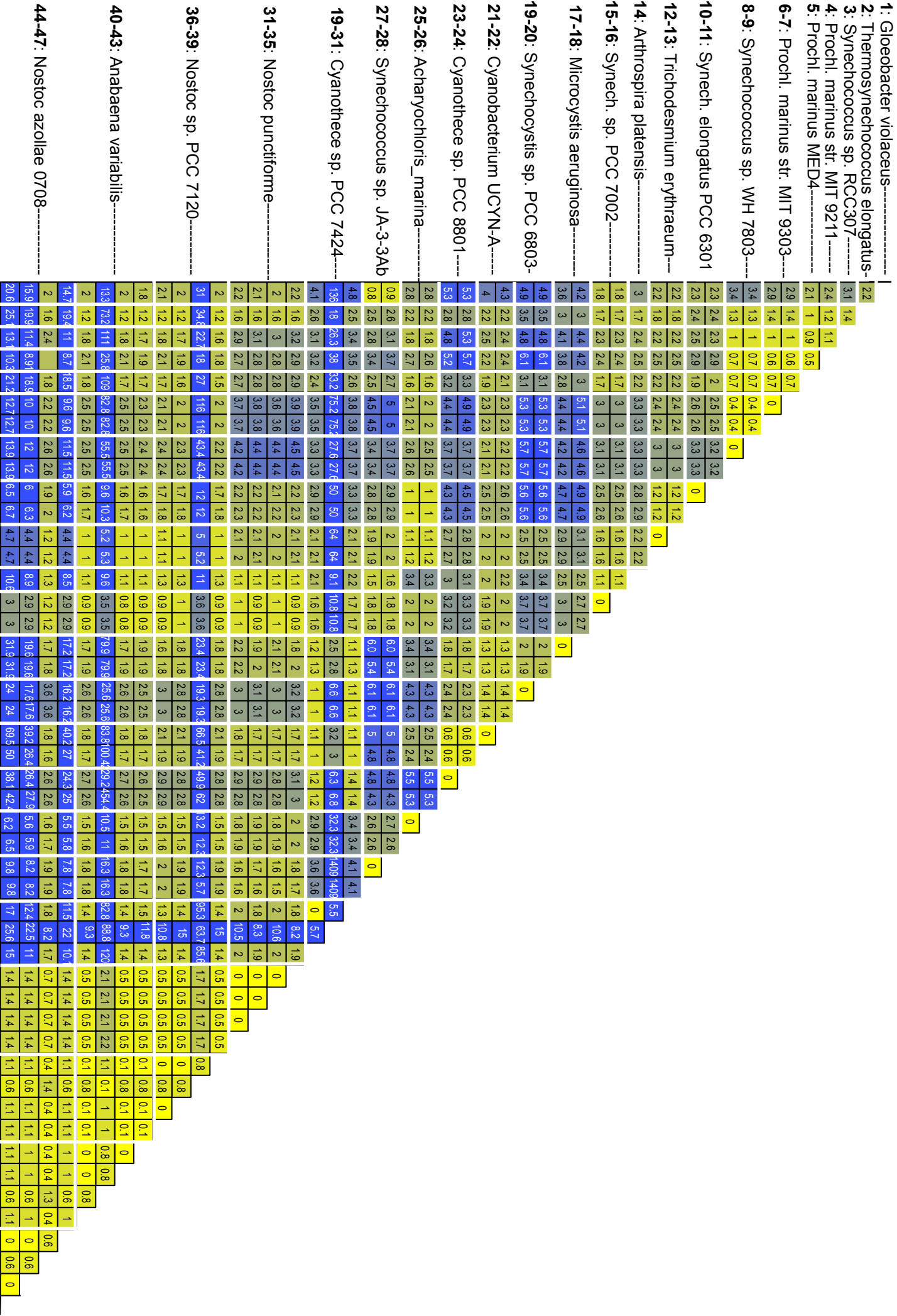

Supplement: Additional file 9 — Distance matrix of cyanobacterial ITS-region. Distance matrix of the internal transcribed spacer sequence region in cyanobacteria. Genetic distances have been estimated according to the K80 substitution model. White lines separate sequence copies of different species. Distances ≥5.7 are displayed by the same blue color. [file 1471-2180-12-177-S9.pdf]
