# Supplementary material for: Gene copy number variation and its significance in cyanobacterial phylogeny
Source: BMC Microbiol. 2012 Aug 15;12:177. doi: 10.1186/1471-2180-12-177 (PMC3552681; doi:10.1186/1471-2180-12-177)
Supplement: Additional file 10 — Data of 16S rRNA gene sequences of the different eubacterial phyla. Species nomenclature, genome sizes, 16S rRNA gene copy numbers and accession numbers from the eubacterial taxa used in this study. [file 1471-2180-12-177-S10.pdf]

species	genome size	16S rRNA copy numbers	accession numbers	species	genome size	16S rRNA copy numbers	accession numbers
**Aquificae**				**Bacteroidetes**			
Aquifex aeolicus VF5	1.6	2	AE000657.1	Alistipes shahii WAL 8301	3.8	1	FP929032
Desulfurobacterium thermolithotrophum DSM 11699	1.5	2	CP002543.1	Bacteroides fragilis NCTC 9343	5.23565	6	CR626927.1
Hydrogenobacter thermophilus TK-6	1.7	1	AP011112.1	Bacteroides helcogenes P 36-108	4	4	CP002352.1
Hydrogenobaculum sp. Y04AAS1	1.6	2	CP001130.1	Bacteroides salanitronis DSM 18170	4.2	6	CP002530.1
Sulfurihydrogenibium sp. YO3AOP1	1.8	3	CP001080.1	Bacteroides thetaiotaomicron VPI-5482	6.29336	5	AE015928.1
Thermocrinis albus DSM 14484	1.5	1	CP001931.1	Bacteroides vulgatus ATCC 8482	5.16319	7	CP000139.1
Thermovibrio ammonificans HB-1	1.8	3	CP002444.1	Blattabacterium sp. (Blattella germanica) str. Bge	0.64	1	CP001487.1
**Chloroflexi**				Candidatus Amoebophilus asiaticus 5a2	1.9	1	CP001102.1
Anaerolinea thermophila UNI-1	3.5	2	AP012029.1	Candidatus Sulcia muelleri CARI	0.28	1	CP002163.1
Chloroflexus aggregans DSM 9485	4.7	3	CP001337.1	Capnocytophaga ochracea DSM 7271	2.6	3	CP001632.1
Chloroflexus aurantiacus J-10-fl	5.3	3	CP000909.1	Cellulophaga algicola DSM 14237	4.9	3	CP002453.1
Chloroflexus sp. Y-400-fl	5.3	3	CP001364.1	Chitinophaga pinensis DSM 2588	9.12735	6	CP001699.1
Dehalococcoides sp. BAV1	1.3	1	CP00688.1	Chlorobaculum parvum NCIB 8327	2.289249	2	CP001099.1
Dehalococcoides ethenogenes 195	1.5	1	CP000027.1	Chlorobium luteolum DSM 273	2.364842	2	CP000096.1
Dehalogenimonas lykanthroporepellens BL-DC-9	1.7	1	CP002084.1	Chlorobium phaeobacteroides DSM 266	3.13390	2	CP000492.1
Herpetosiphon aurantiacus ATCC 23779	6.8	5	CP000875.1	Chlorobium phaeovibrioides DSM 265	1.966858	1	CP000607.1
Roseiflexus sp. RS-1	5.8	2	CP000686.1	Chlorobium tepidum TLS	2.154946		AE006470.1
Roseiflexus castenholzii DSM 13941	5.7	2	CP000804.1	Chloroherpeton thalassium ATCC 35110	3.293456	1	CP001100.1
Sphaerobacter thermophilus DSM 20745	4.0	1	CP001823.1	Cytophaga hutchinsonii ATCC 33406	4.433218	3	CP000383.1
Thermomicrobium roseum DSM 5159	2.9	1	CP001275.1	Dyadobacter fermentans DSM 18053	6.96779	4	CP001619.1
**Spirochaetae**				Flavobacterium johnsoniae UW101	6.096872	6	CP000685.1
Borellia afzelii PKo	1.23783	1	CP000395.1	Fluviicola taffensis DSM 16823	4.633577	2	CP002542.1
Borrelia burgdorferi B31	1.51913	1	AE000783.1	Gramella forsetii KT0803	3.798465		CU207366.1
Borrelia duttonii Ly	1.567	1	CP000976.1	Haliscomenobacter hydrossis DSM 1100	8.371686	2	CP002691.1
Borrelia garinii PBi	0.982668		CP000013.1	Krokinobacter sp. 4H-3-7-5	3.389993	3	CP002528.1
Borrelia hermsii DAH	1.059 5	1	CP000048.1	Lacinutrix sp. 5H-3-7-4	3.296168	2	CP002825.1
Borrelia recurrentis A1	0.930981	1	CP000993.1	Leadbetterella byssophila DSM 17132	4.059653		CP002305.1
Borrelia turicatae 91E135	0.92	1	CP000049.1	Maribacter sp. HTCC2170	3.868304	2	CP002157.1
Brachyspira hyodysenteriae WA1	3.036	1	CP001357.1	Marivirga tractuosa DSM 4126	4.511574	2	CP002349.1
Brachyspira murdochii DSM 12563	3.2	1	CP001959.1	Odoribacter splanchnicus DSM 20712	4.392288	4	CP002544.1
Brachyspira pilosicoli 95/1000	2.6	1	CP002025.1	Paludibacter propionicigenes WB4	3.685504	3	CP002345.1
Leptospira biflexa serovar Patoc strain	3.603977	2	CP000777.1	Parabacteroides distasonis ATCC 850	4.811379	7	CP000140.1
Leptospira borgpetersenii serovar Hardjo-bovis JB197	3.87647	2	CP000350.1	Pedobacter heparinus DSM 2366	5.167383	3	CP001681.1
Leptospira interrogans serovar Lai str. 56601	4.69224	2	AE010300.2	Pelodictyon phaeoclathratiforme BU-1	3.018238	2	CP001110.1
Spirochaeta coccoides DSM 17374	2.2	3	CP002659.1	Porphyromonas gingivalis ATCC 33277	2.354886	4	AP009380.1
Spirochaeta smaragdinae DSM 11293	4.7	2	CP002116.1	Prevotella denticola F0289	2.937589	4	CP002589.1
Spirochaeta sp. Buddy	3.3	4	CP002541.1	Prevotella ruminicola 23	3.61955 9	4	CP002006.1
Spirochaeta thermophila DSM 6192	2.5	2	CP001698.1	Prosthecochloris aestuarii DSM 271	2.512923	1	CP001108.1
Treponema azotonutricium ZAS-9	3.855671	2	CP001841.1	Rhodothermus marinus DSM 4252	3.261604	1	CP001807.1
Treponema brennaborense DSM 12168	3.1	3	CP002696.1	Riemerella anatipestifer DSM 15868	2.155121	3	CP002346.1
Treponema denticola ATCC 35405	2.84	2	AE017226.1	Robiginitalea biformata HTCC2501	3.530383	2	CP001712.1
Treponema pallidum subsp. pallidum SS14	1.14	2	CP000805.1	Runella slithyformis DSM 19594	6.568739	2	CP002859.1
Treponema paraluiscuniculi Cuniculi A	1.1	2	CP002103.1	Salinibacter ruber DSM 13855	3.551823	1	CP000159.1
Treponema primitia ZAS-2	4.1	2	CP001843.1	Sphingobacterium sp. 21	6.226409	4	CP002584.1
Treponema succinifaciens DSM 2489	2.87	4	CP002631.1	Spirosoma linguale DSM 74	8.078757	4	CP001769.1
				Weeksella virosa DSM 16922	2.272954	5	CP002455.1
				Zunongwangia profunda SM-A87	5.128187	3	CP001650.1
